# Myricetin-bound crystal structure of the SARS-CoV-2 helicase NSP13 facilitates the discovery of novel natural inhibitors

**DOI:** 10.1107/S2059798325004498

**Published:** 2025-05-27

**Authors:** Patrick Kloskowski, Piotr Neumann, Priya Kumar, Annette Berndt, Matthias Dobbelstein, Ralf Ficner

**Affiliations:** a Department of Molecular Structural Biology, Institute of Microbiology and Genetics, Göttingen Center of Molecular Biosciences (GZMB) University of Göttingen Justus-von-Liebig-Weg 11 37077Göttingen Germany; b Cluster of Excellence ‘Multiscale Bioimaging: From Molecular Machines to Networks of Excitable Cells’ (MBExC) University of Göttingen 37075Göttingen Germany; c Department of Molecular Oncology, Göttingen Center of Molecular Biosciences (GZMB) University Medical Center Göttingen Justus-von-Liebig-Weg 11 37077Göttingen Germany; F. Hoffmann-La Roche Ltd, Switzerland

**Keywords:** SARS-CoV-2, NSP13 helicase, myricetin, virtual screening, natural inhibitors, caffeic acid derivatives, antiviral drug development

## Abstract

The SARS-CoV-2 helicase NSP13 is a highly conserved and essential component of the viral replication machinery, making it a promising target for antiviral drug development. Here, we present the 2 Å resolution crystal structure of NSP13 bound to the natural flavonoid myricetin, revealing a conserved allosteric binding site. Guided by these structural findings, a virtual screening campaign identified the caffeic acid derivatives rosmarinic acid and chlorogenic acid as potential novel natural inhibitors, which were experimentally validated to inhibit RNA-unwinding activity. This study provides structural insights that could support ongoing drug-discovery efforts targeting NSP13 in SARS-CoV-2 and other coronaviruses with pandemic potential.

## Abstract 2

The myricetin-bound crystal structure of the SARS-CoV-2 helicase reveals a conserved allosteric binding site. Guided by these structural insights, caffeic acid derivatives identified through virtual screening were experimentally validated as novel natural inhibitors of NSP13.

## 1. Introduction

Natural products, particularly secondary metabolites from bacteria, fungi and plants, are renowned for their remarkable chemical diversity and their pivotal role in drug discovery and development. Nearly half of the drugs approved between 1981 and 2019 originated from natural sources, underscoring their significant contributions to various therapeutic areas (Newman & Cragg, 2020 ▸). In response to the COVID-19 pandemic, natural products have gained increased attention as potential lead compounds against SARS-CoV-2 (Ebob *et al.*, 2021 ▸). Among these, myricetin, a flavonoid that is abundant in fruits, vegetables and teas, has emerged as a particularly promising lead compound.

The activity of myricetin against coronaviruses was first identified during the SARS epidemic, which occurred from 2002 to 2003. Research by Yu *et al.* (2012 ▸) demonstrated that myricetin inhibits the ATPase activity of the SARS-CoV-1 helicase nonstructural protein 13 (NSP13). During the COVID-19 pandemic, myricetin was also considered for therapeutic use and was shown to inhibit other key SARS-CoV-2 proteins, including the main protease (M^pro^; NSP5), spike protein (S protein) and RNA-dependent RNA polymerase (RdRp; NSP12) (Su *et al.*, 2021 ▸; Pan *et al.*, 2023 ▸; Kuzikov *et al.*, 2024 ▸). Moreover, cell-based studies demonstrated that the flavonoid reduced SARS-CoV-2 infection and replication, exhibited anti-inflammatory properties and showed low cytotoxicity (Su *et al.*, 2021 ▸; Xiao *et al.*, 2021 ▸; Pan *et al.*, 2023 ▸). Myricetin was also re-evaluated as a helicase inhibitor, given the 99.8% sequence identity between the orthologs from SARS-CoV-1 and SARS-CoV-2 (Halma *et al.*, 2022 ▸). More recent investigations revealed that myricetin affects not only the ATPase but also the RNA-unwinding activity of NSP13 (Corona *et al.*, 2022 ▸; Inniss *et al.*, 2024 ▸; Kuzikov *et al.*, 2024 ▸). However, these findings contrast with the earlier work by Yu *et al.* (2012 ▸), who reported ATPase inhibition alone, highlighting the need to determine the binding site of myricetin in order to clarify its mechanism of action and guide future NSP13-targeted inhibitor development.

In response to the SARS-CoV-2 pandemic, NSP13 emerged as an attractive target, prompting multiple high-throughput, virtual and fragment-based screening campaigns, as reviewed by Mehyar (2023 ▸). Recent studies highlight that the discovery of novel NSP13 inhibitors remains an active area of research in numerous laboratories (Otsuka *et al.*, 2024 ▸; Zhang *et al.*, 2024 ▸; Soper *et al.*, 2024 ▸; Di Paco *et al.*, 2024 ▸; Ramsey *et al.*, 2024 ▸; Martin *et al.*, 2024 ▸). NSP13 is an Upf1-like helicase belonging to superfamily 1B that is responsible for unwinding nucleic acids in an NTP-dependent manner with 5′- to 3′- polarity. Mutagenesis experiments have confirmed that NSP13 is a critical component of SARS-CoV-2 and other coronaviruses (Grimes & Denison, 2024 ▸). Its significance is underscored by its high sequence conservation with SARS-CoV-1 NSP13 (99.8% identity) and MERS-CoV NSP13 (70% identity), as well as its strong structural homology to these orthologues, with *DALIZ*-scores of 42.1 or higher (Holm *et al.*, 2023 ▸). This remarkable conservation across coronaviruses highlights its potential as a pan-coronavirus antiviral target. Reinforcing its significance, several structures of SARS-CoV-2 NSP13 have been resolved using X-ray crystallography and cryo-electron microscopy, providing valuable insights into its role in the viral life cycle and the associated conformational changes (Yan, Ge *et al.*, 2021 ▸; Yan, Yang *et al.*, 2021 ▸; Malone *et al.*, 2021 ▸; Newman *et al.*, 2021 ▸; Chen *et al.*, 2020 ▸, 2022 ▸). However, no experimentally determined structures of NSP13 in complex with inhibitors have yet been reported. Such structures are crucial for understanding the binding sites and molecular interactions, which are essential for rational drug design. The absence of inhibitor-bound structures represents a significant gap in the ongoing efforts to develop therapeutics targeting NSP13.

In this study, we present the crystal structure of myricetin-bound SARS-CoV-2 NSP13, revealing the binding site of myricetin for the first time and providing crucial insights into its mechanism of inhibition. Leveraging this structural information, along with fragment-screening data from Newman *et al.* (2021 ▸), virtual screening identified novel natural inhibitors, including rosmarinic acid and chlorogenic acid. Both compounds, which are derivatives of caffeic acid, were biochemically validated as NSP13 inhibitors. These findings may support the development of therapeutics against SARS-CoV-2 and other emerging coronaviruses by targeting NSP13.

## 2. Experimental procedures

### 2.1. Protein production and purification

Production and purification of NSP13 from SARS-CoV-2 (NCBI Accession YP_009725308) were performed using a modified protocol based on Newman *et al.* (2021 ▸). A codon-optimized synthetic gene for SARS-CoV-2 NSP13 (BioCat) was cloned into a pET-52b(+) vector (Merck), which was modified to replace its original cleavage site with a TEV protease cleavage site. The resulting plasmid was transformed into *Escherichia coli* Rosetta2(DE3) cells, which were cultivated in Terrific broth and induced with 300 µ*M* isopropyl β-d-1-thiogalactoyranoside to produce N-terminally Strep II-tagged SARS-CoV-2 NSP13.

Harvested cells were resuspended in a lysis buffer consisting of 50 m*M* HEPES pH 7.5, 500 m*M* NaCl, 5% glycerol, 0.5 m*M* TCEP supplemented with a cOmplete protease-inhibitor cocktail (Roche). Cell disruption was achieved using an LM10 microfluidizer (Microfluidics) and the clarified lysate was loaded onto a pre-equilibrated StrepTrap XT column (Cytiva). To remove nucleic acids bound to NSP13, lysis buffer supplemented with 2 *M* LiCl was applied.

NSP13 was eluted using lysis buffer containing 40 m*M* biotin and then incubated overnight with TEV protease at 4°C to remove the tag. The digested NSP13 was further purified by size-exclusion chromatography on a Superdex 200 16/60 column (Cytiva) equilibrated with 50 m*M* HEPES pH 7.5, 500 m*M* NaCl, 0.5 m*M* TCEP. The purified NSP13 was then concentrated to 20 mg ml^−1^.

### 2.2. Protein crystallization, ligand co-crystallization and ligand soaking

Protein crystals of phosphate-bound SARS-CoV-2 NSP13 (PDB entry 6zsl) were obtained using the procedure described by Newman *et al.* (2021 ▸), as summarized in Table 1 ▸. Crystallization was performed at 293 K using the sitting-drop vapour-diffusion method combined with a microseeding protocol. The crystallization conditions included 16% ethylene glycol, 8% PEG 8000, 0.05 *M* HEPES, 0.05 *M* MOPS, 0.03 *M* sodium nitrate, 0.03 *M* sodium phosphate, 0.03 *M* ammonium sulfate and were based on Morpheus Screen condition C6 (Molecular Dimensions, catalogue No. MD1-47). The purified protein was diluted fourfold in water to 5 mg ml^−1^, while the reservoir solution was prepared exclusively from stock solutions [catalogue Nos. MD2-100(250)-82, MD2-100(250)-72 and MD2-100(250)-101]. A Mosquito Xtal3 robot (SPT Labtech) sequentially dispensed 250 nl protein solution, 33 nl microseeds and 250 nl reservoir solution into each well of an MRC 96-well 3-lens plate (SWISSCI).

The reproduction of well diffracting crystals using the initial crystallization conditions was unsuccessful. However, the addition of 3% MPD significantly improved the crystal quality, as identified using the Additive Screen (Hampton Research, catalogue No. HR2-428). Subsequent fine screening revealed that 9% MPD (Merck, catalogue No. 68346) produced the best-diffracting crystals, consistently achieving a resolution of approximately 2 Å. These optimized crystals exhibited slight changes in unit-cell dimensions and solvent content compared with the initial conditions, as determined by comparing PDB entries 6zsl and 9i4v (Δ*a*, +0.26%; Δ*b*, +0.81%; Δ*c*, +1.97%; Δα, +1.20%; Δβ, −0.43%; Δγ, −0.59%; solvent content, +4.89%). All structures reported in this study were derived from crystals grown under the optimized conditions including 9% MPD.

Both co-crystallization and soaking methods were used to obtain crystals of NSP1 in complex with myricetin (Tokyo Chemical Industry, catalogue No. M2131), rosmarinic acid (Cayman Chemical, catalogue No. 70900) and chlorogenic acid (Cayman Chemical, catalogue No. 70930). For co-crystallization, the protein was incubated with each ligand at a fivefold to 20-fold molar excess at room temperature (RT) for 30–60 min prior to crystallization. In the soaking method, pre-grown crystals were exposed to ligand concentrations ranging from 5 to 50 m*M* for 30 min to 2 h. A total of 40 crystals, including unliganded and putative ligand-bound forms, were successfully obtained. Before flash-cooling, each crystal was cryoprotected using reservoir solution with increased concentrations of ethylene glycol and PEG 8000 (Table 1 ▸).

### 2.3. Data collection, structure determination and ligand identification

Diffraction images of unliganded and putative ligand-bound SARS-CoV-2 NSP13 crystals were collected on EMBL beamline P13 at PETRA III, DESY, Hamburg, Germany and processed using *autoPROC* (Vonrhein *et al.*, 2011 ▸), which integrates *XDS*, *POINTLESS*, *AIMLESS* and *CCP*4 (Kabsch, 2010 ▸; Evans, 2006 ▸; Evans & Murshudov, 2013 ▸; Winn *et al.*, 2011 ▸). In total, 40 data sets were collected: 18 from ligand-bound crystals, comprising three data sets each from co-crystallization and soaking experiments with myricetin, rosmarinic acid and chlorogenic acid (*i.e.* six data sets per compound), and 22 from unliganded crystals. All data sets exhibited similar resolution, data quality and unit-cell dimensions, consistent with the statistics reported for the deposited structures (Table 2 ▸).

The structure determination was performed using *DIMPLE* (Wojdyr *et al.*, 2013 ▸), a macromolecular crystallography pipeline for refinement and ligand screening, which uses programs from the *CCP*4 suite (Murshudov *et al.*, 2011 ▸; Agirre *et al.*, 2023 ▸). Previously published SARS-CoV-2 NSP13 structures, including PDB entries 6zsl, 5rob, 7nio and 7nn0 (Newman *et al.*, 2021 ▸), were used as initial search models for molecular replacement. *DIMPLE* detected unmodelled electron density at the 5′ end of the RNA-binding channel site in both crystal forms, located in each of the two monomers (chains *A* and *B*) within the asymmetric unit. This density, attributed to the reservoir buffer component MOPS (Table 3 ▸; Supplementary Fig. S1), corresponds to a region that is known to interact with RNA (PDB entry 7kro) and fragments (PDB entries 5rlz and 5rmm). No additional significant electron density was observed.

**Figure 1:**
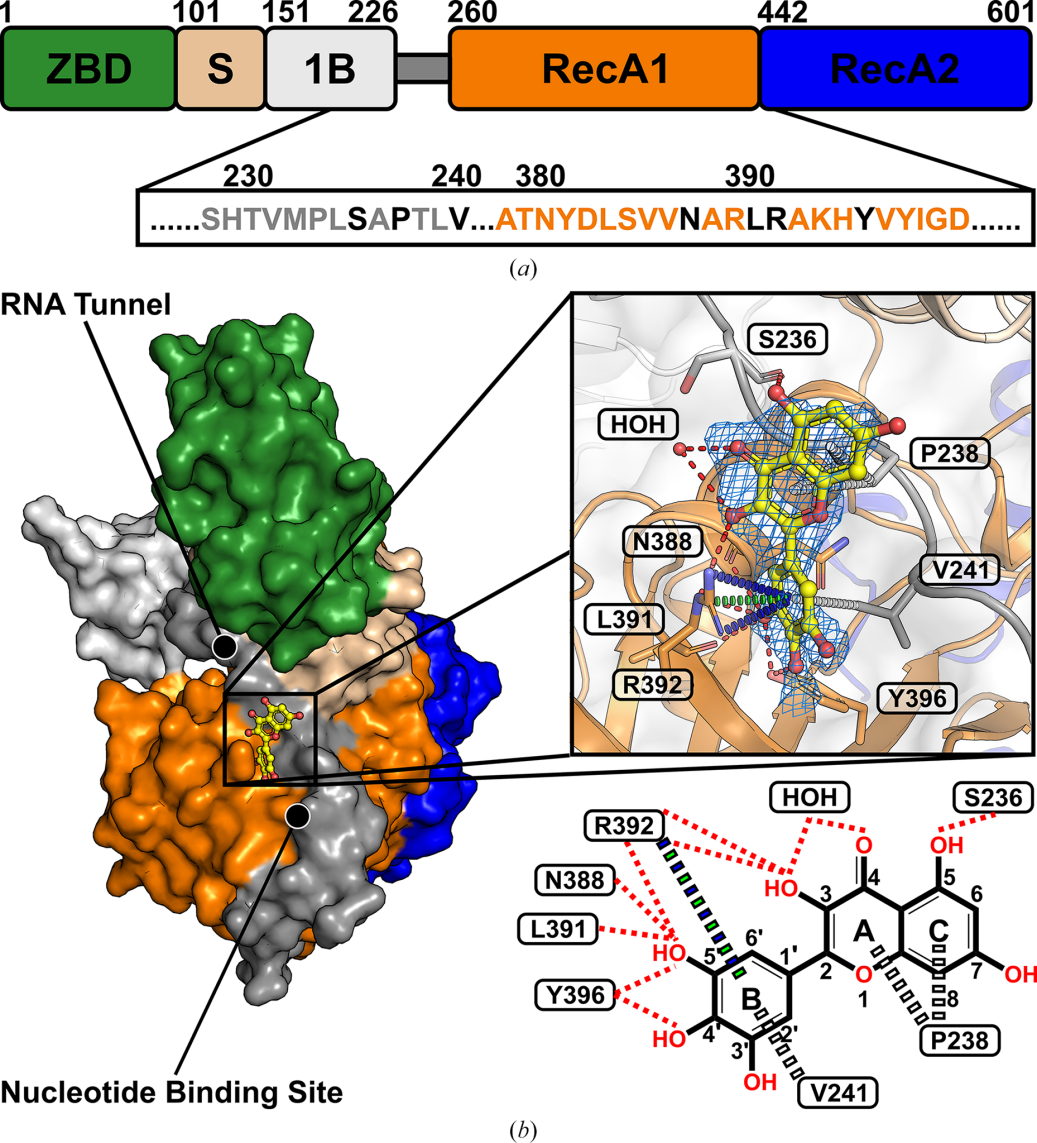
Myricetin-bound crystal structure of SARS-CoV-2 NSP13. (*a*) Domain organization of NSP13, showing the zinc-binding domain (ZBD; green), stalk domain (wheat), 1B domain (grey), RecA1 domain (orange), RecA2 domain (blue) and the linker connecting the 1B and RecA1 domains (dark grey). Below, a segment of the RecA1 domain and linker highlights the myricetin-interacting amino acids (black). (*b*) Surface representation of NSP13, colour-coded as in (*a*), with myricetin displayed as a yellow ball-and-stick model. The positions of the nucleotide-binding site and RNA-binding channel are indicated. The inset shows the binding site with interacting residues (sticks), a water molecule (sphere), hydrogen bonds (red dashed lines), carbon–π interactions (white dashed lines), cation–π interactions (green dashed lines) and donor–π interactions (blue dashed lines). The polder OMIT *mF*_o_ − *DF*_c_ electron-density map for myricetin is displayed as a blue mesh, contoured at 4σ. A residual density near the 4′-hydroxyl group is likely to correspond to an alternative water molecule, but it was not included in the model because it fell below the refinement cutoff (map r.m.s.d. < 0.9 e Å^−3^). The bottom right panel illustrates the chemical structure of myricetin and its interaction network with key NSP13 residues, including hydrogen bonds (red dashed lines), carbon–π interactions (white dashed lines) and cation–π and donor–π interactions (blue–green dashed lines).

To further investigate, the refined structural models were analysed using *PanDDA*, a tool designed to detect low-occupancy ligands across multiple crystallographic data sets (Pearce *et al.*, 2017 ▸). *PanDDA* analysis revealed an additional region of excess electron density in the *mF*_o_ − *DF*_c_ electron-density map at the +3σ level in three myricetin-soaked NSP13 crystal structures. This density, which was not detected by *DIMPLE*, was located in chain *B* on the surface of the RecA1 domain near its interface with the stalk domain and was attributed to myricetin based on its shape (Table 3 ▸; Fig. 1 ▸).

Each atomic model underwent manual rebuilding in *Coot* (Emsley *et al.*, 2010 ▸), alternating with cycles of reciprocal-space and real-space refinement using a *Phenix*-based refinement pipeline (Garbers *et al.*, 2024 ▸). As part of the solvent-modelling procedure, only water molecules with map r.m.s.d. values of 0.9 e Å^−3^ or higher were retained. Besides, several loop regions that lacked sufficient electron density for reliable modelling were omitted from the final structures, including residues 95–102, 186–193 and 203–206 in chain *A* and residues 204–207 and 337–339 in chain *B*.

Best unliganded and myricetin-bound models were selected based on resolution, *R* factor and, where applicable, ligand occupancy and RSCC fit. The refined structures have been deposited in the Protein Data Bank, with the myricetin-bound structure assigned PDB code 9i1s and the unliganded structure assigned PDB code 9i4v. Data-collection and processing statistics are provided in Table 2 ▸ and refinement statistics are summarized in Table 4 ▸. Figures were prepared using the open-source version of *PyMOL* (version 2.6; Schrödinger).

### 2.4. Myricetin binding-site analysis

#### 2.4.1. *In silico* assessment of the binding affinities of myricetin moieties

The individual contributions of the myricetin moieties to its overall binding affinity with NSP13 were computationally assessed, informed by the approach described by Neumann *et al.* (2024 ▸). The pyrogallol and trihydroxychromone moieties (chemical structures shown in Table 3 ▸) were extracted from the crystallographic binding pose of myricetin and rescored in place using the *Gnina* docking software with both Vina and Vinardo scoring functions (Trott & Olson, 2010 ▸; Quiroga & Villarreal, 2016 ▸). No conformational sampling was performed, as the goal was to estimate the individual contributions of each moiety to binding in the crystallographic context. These results were subsequently used to prioritize energetically favourable substructures for the virtual screening campaign targeting NSP13.

#### 2.4.2. Interaction analysis

Intermolecular interactions between myricetin and SARS-CoV-2 NSP13 were analysed using the *Arpeggio* webserver (https://biosig.lab.uq.edu.au/arpeggioweb/; Jubb *et al.*, 2017 ▸) to gain a detailed understanding of the myricetin-binding site and guide the virtual screening campaign. Key interactions, including hydrogen bonds, carbon–π interactions, cation–π interactions and donor–π interactions between myricetin and NSP13, were identified. These interactions were used to impose constraints on the virtual screening process, prioritizing ligands capable of replicating these essential inter­actions with NSP13.

#### 2.4.3. Sequence-conservation analysis

Residues interacting with myricetin were evaluated for sequence conservation to determine whether the myricetin binding site on NSP13 could serve as a viable target across betacoronaviruses, including SARS-CoV-1 and MERS-CoV. The sequence conservation was assessed using the protocol outlined by Newman *et al.* (2021 ▸) through a similarity search for NSP13 (NCBI Accession YP_009725308) in the UniProt database (Bateman *et al.*, 2023 ▸). Default search settings were applied, with additional filters restricting results to beta­coronaviruses (Taxon ID 694002) and including only reviewed entries (Swiss-Prot). The results were visualized using a logo plot generated with *WebLogo* (version 2.8.2; https://weblogo.berkeley.edu/; Crooks *et al.*, 2004 ▸; Schneider & Stephens, 1990 ▸). The information was incorporated into the virtual screening campaign to identify ligands with potential inhibitory activity across betacoronaviruses, including SARS-CoV-2.

### 2.5. Virtual screening of inhibitors against SARS-CoV-2 NSP13

In order to identify novel pyrogallol-based inhibitors of SARS-CoV-2 NSP13, a combined virtual screening strategy was employed, following the approach outlined by Garbers *et al.* (2024 ▸). The workflow began with a ligand-based screening, followed by structure-based refinement. Initial screening was conducted using *ROCS* (v3.6.0.1; Hawkins *et al.*, 2007 ▸) from the *OpenEye* software suite (https://www.eyesopen.com/; free academic licence). *ROCS* superimposed database molecules onto a myricetin-based query using shape and chemical feature alignment. Its high efficiency enabled the rapid screening of extensive compound libraries, processing hundreds of molecules per second on a single CPU. Promising hits from *ROCS* were re-evaluated using the *OpenEye*molecular-docking tool *HYBRID* (version 4.2.1.1; McGann, 2011 ▸, 2012 ▸). *HYBRID* refined the selection by scoring the binding pose of each molecule within the myricetin binding site on NSP13. To ensure robust candidate selection, *HYBRID* results were validated by redocking with *Gnina* (version 1.1; McNutt *et al.*, 2021 ▸). Candidates that performed strongly across both docking programs were prioritized as potential NSP13 inhibitors. The entire screening process is illustrated schematically in Fig. 5.

#### 2.5.1. Ligand database preparation

For this study, the DrugBank database (version 5.1.12; Knox *et al.*, 2024 ▸) was used, containing 9716 three-dimensional structures of approved, investigational, experimental and nutraceutical compounds. The database was curated for virtual screening through preprocessing steps, including filtering, tautomerization and normalization of each structure using the *OpenEye* tools *FILTER* (version 4.2.2.1; Hawkins *et al.*, 2010 ▸) and *Tautomers* (version 2.2.2.1; https://www.eyesopen.com/quacpac). To ensure a comprehensive representation of possible shapes and chemical feature distributions, conformer generation was performed using the *OpenEye* tool *OMEGA* (version 4.2.2.1; Hawkins *et al.*, 2010 ▸). Up to 500 conformers per ligand were generated, resulting in a final database of 1 993 335 conformers for use in *ROCS*.

#### 2.5.2. *ROCS* query preparation

*ROCS* (*Rapid Overlay of Chemical Structures*) is a ligand-based virtual screening tool that identifies potential inhibitors by superimposing database molecules onto a query using shape and chemical feature alignment.

**Figure 2:**
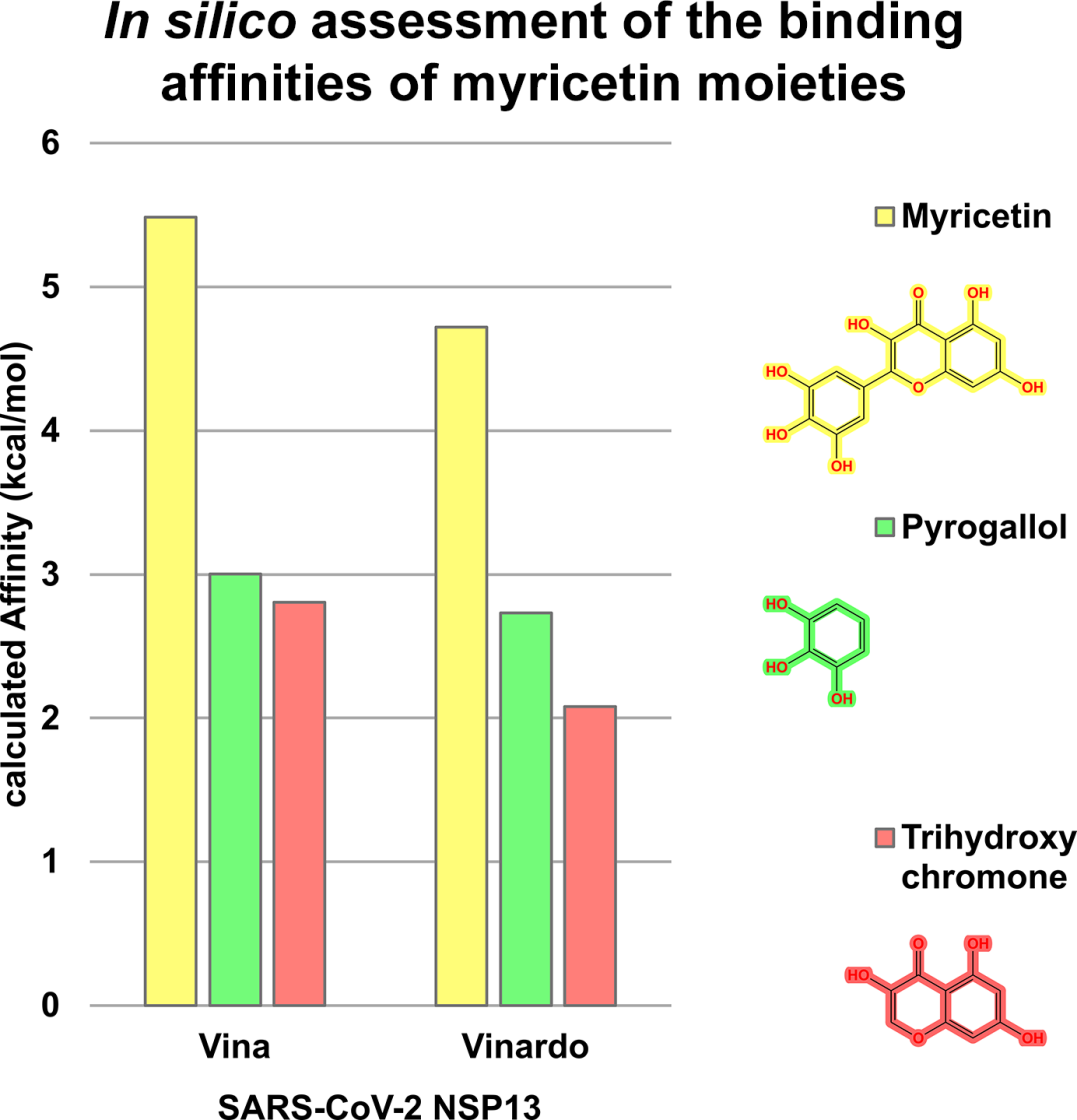
*In silico* assessment of the binding affinities of myricetin moieties to SARS-CoV-2 NSP13. The binding affinities of myricetin (yellow), its pyrogallol moiety (green) and its trihydroxychromone moiety (red) were computationally evaluated against SARS-CoV-2 NSP13 using the *Gnina* docking tool, with both the Vina and Vinardo scoring functions. Binding affinities (kcal mol^−1^) were calculated based on the positions of each moiety within the myricetin binding site. The pyrogallol moiety consistently exhibited stronger binding affinities compared with the tri­hydroxychromone moiety, highlighting its significant contribution to the interaction of myricetin with NSP13.

The initial *ROCS* query was derived from the shape and chemical feature distribution of the pose of myricetin bound to SARS-CoV-2 NSP13. Key features, including hydrogen-bond donors, hydrogen-bond acceptors and aromatic rings, were incorporated to reflect the conserved interactions identified between myricetin and NSP13. Particular emphasis was placed on the pyrogallol moiety, which contributed most significantly to binding (Fig. 2 ▸). This moiety was prioritized by upweighting its associated chemical features, including two hydrogen-bond acceptors, two hydrogen-bond donors and an aromatic ring, as shown in the query depicted in Fig. 5.

However, the shape of the initial query disfavoured the identification of larger molecules exceeding the size of myricetin. To address this limitation, fragments identified in the crystallographic fragment screen by Newman *et al.* (2021 ▸) were used to extend the query. Fragment-bound structures were superimposed onto the myricetin-bound structure, revealing that fragments RYM, VVM and VVY, derived from PDB entries 5rme, 5rlc and 5rld, respectively, overlapped with or were adjacent to myricetin. These fragments were analysed for their interactions with SARS-CoV-2 NSP13, and the sequence conservation of interacting residues was assessed as described in Sections 2.4.2 and 2.4.3.

Based on these findings, a new ligand ensemble was constructed to generate an extended query. This query incorporated the shape and chemical feature distribution of all constituent molecules, ensuring that the chemical features captured all conserved interactions identified between myricetin, the fragments and NSP13. At the same time, the query maintained a focus on the key chemical features of the pyrogallol moiety, consistent with the initial query. The extended query (Supplementary Fig. S6) was used in a *ROCS* search against the prepared DrugBank database. Default settings were applied and the top 500 hits, ranked by the Tanimoto Combo score (Hawkins *et al.*, 2007 ▸), were retained.

#### 2.5.3. Molecular docking of *ROCS* results with *HYBRID*

*ROCS* results were further refined using molecular docking performed with the *OpenEyeHYBRID* tool, as described by Garbers *et al.* (2024 ▸). *HYBRID* is a ligand-guided docking tool that integrates information from both the receptor and a bound ligand to enhance virtual screening. It systematically explores ligand conformers within the binding site, narrowing the search space based on shape and chemical complementarity to the reference ligand.

During docking, both the ligand conformer and the protein structure are treated as rigid. Ligand flexibility was implicitly accounted for by generating up to 500 conformers for each *ROCS* hit using *OMEGA*. To address potential protein flexibility, *HYBRID* supports the use of multiple receptor conformers, such as those derived from myricetin-bound and fragment-bound NSP13 structures. However, an analysis of side-chain conformations interacting with myricetin and selected fragments revealed no significant structural differences (Supplementary Fig. S5). Pairwise structure alignment yielded all-atom r.m.s.d. values not exceeding 0.32 Å. Consequently, only the myricetin-bound NSP13 structure was used as the receptor (PDB entry 9i1s).

*HYBRID* was executed in high-resolution mode, generating one pose per ligand conformer. The top 500 ligand poses, ranked by the ChemGauss4 score (McGann, 2012 ▸), were retained.

#### 2.5.4. Re-evaluation of *HYBRID* results with *Gnina*

The protocol described by Garbers *et al.* (2024 ▸) utilized *Gnina* to refine potential lead candidates by selecting those that performed well in both *HYBRID* and *Gnina* docking experiments. *Gnina* is a structure-based docking tool that employs convolutional neural networks (CNNs) to predict and score protein–ligand binding poses. By re-evaluating output poses, *Gnina* has demonstrated superior performance in redocking and cross-docking tasks, especially when the binding pocket is well defined.

Ligand poses determined by *HYBRID* were reassessed on the myricetin-bound structure of NSP13. Each docking experiment utilized an ensemble of CNNs in combination with either the hybrid scoring function Vina (Trott & Olson, 2010 ▸) or the empirical scoring function Vinardo (Quiroga & Villarreal, 2016 ▸). Default settings were applied, except for the exhaustiveness parameter, which was increased from 8 to 64 to enhance docking accuracy, albeit with higher computational cost.

*Gnina* generated up to nine conformers per ligand, each ranked by its CNN score. For each docking experiment, the CNN score, CNN affinity and calculated binding affinity were reported to comprehensively evaluate the binding performance of each ligand.

### 2.6. Enzymatic assays

Enzymatic assays were conducted following the protocol of Corona *et al.* (2022 ▸). Although NSP13 is assumed to target RNA *in vivo*, enzymatic characterization has also revealed its affinity for DNA (Jang *et al.*, 2020 ▸; Mickolajczyk *et al.*, 2021 ▸). This property allows the use of DNA substrates instead of RNA. Both assays were used to evaluate the effects of myricetin (Tokyo Chemical Industry, catalogue No. M2131), rosmarinic acid (Cayman Chemical, catalogue No. 70900) and chlorogenic acid (Cayman Chemical, catalogue No. 70930) on SARS-CoV-2 NSP13. Raw values were first background-corrected by subtracting the solvent-only negative control and then normalized to the DMSO-only positive control, which was set to 100% activity. Normalized data were subsequently analysed using an online IC_50_ calculator (AAT Bioquest; https://www.aatbio.com/tools/ic50-calculator) to determine IC_50_ values and generate plots for each experiment.

#### 2.6.1. Unwinding assay

NSP13 unwinding activity was measured using a fluorescence-based assay in black 96-well plates (Corning, catalogue No. 3993) with a total volume of 80 µl. The reaction mixture comsisted of 20 m*M* Tris–HCl pH 7.2, 50 m*M* NaCl, 2 m*M* MgCl_2_, 0.5 m*M* TCEP, 2 µ*M* Hel Capture oligo (5′-TGG TGC TCG AAC AGT GAC-3′; Eurofins), 5% DMSO or inhibitor and 3 n*M* purified SARS-CoV-2 NSP13.

Following a 10 min pre-incubation of NSP13 with the inhibitor at room temperature (RT), the reaction was initiated by adding 1 m*M* ATP and 750 n*M* annealed DNA substrate. The substrate consisted of the oligonucleotides 5′-AGT CTT CTC CTG GTG CTC GAA CAG TGA C BBQ650-3′ and 5′-CY5 GTC ACT GTT CGA GCA CCA CCT CTT CTG A-3′ (Eurofins). Fluorescence measurements were taken at RT (excitation at 640 nm, emission at 685 nm) using a Victor Nivo plate reader (PerkinElmer).

#### 2.6.3. ATPase assay

NSP13 ATPase activity was assessed using an absorbance-based assay in transparent 96-well plates (Sarstedt, catalogue No. 82.1581001) with a total reaction volume of 50 µl. The reaction mixture included 20 m*M* Tris–HCl pH 7.2, 50 m*M* NaCl, 2 m*M* MgCl_2_, 0.5 m*M* TCEP, 5% DMSO or inhibitor and 30 n*M* purified SARS-CoV-2 NSP13.

The reaction was initiated by adding 400 µ*M* ATP and was incubated at RT for 30 min. Following this, 50 µl Biomol Green Reagent (Enzo Life Sciences) was added and the mixture was incubated for 10 min at RT, protected from light. Absorbance was measured at 650 nm using a Victor Nivo plate reader (PerkinElmer).

### 2.7. Infection assay

#### 2.7.1. Cell culture

Vero E6 cells (Vero C1008) were obtained from the German Primate Centre (DPZ), Göttingen, Germany and served as host cells for SARS-CoV-2 infection. The cells were cultured in Dulbecco’s Modified Eagle’s Medium (DMEM) with GlutaMAX (Gibco) supplemented with 10% fetal bovine serum (FBS; Merck), 200 µ*M*l-glutamine, 50 U ml^−1^ penicillin (Gibco) and 50 U ml^−1^ streptomycin (Gibco). The cells were incubated in a humidified atmosphere at 37°C with 5% CO_2_.

#### 2.7.2. Treatments and SARS-CoV-2 infection

A Wuhan-like SARS-CoV-2 strain, isolated in Göttingen in March 2020 (Stegmann *et al.*, 2021 ▸), was used for infection.

Vero E6 cells (3500 cells per well) were seeded into 96-well plates in medium supplemented with 10% fetal bovine serum (FBS). After 24 h, the medium was replaced with medium containing 2% FBS and a serial dilution of either rosmarinic acid (Cayman Chemical, catalogue No. 70900; 200→6.25 µ*M*), chlorogenic acid (Cayman Chemical, catalogue No. 70930; 200→6.25 µ*M*) or β-d-*N*4-hydroxycytidine (NHC/EIDD-1931; Cayman Chemical, catalogue No. 9002958; 10→0.3125 µ*M*). The cells were incubated with the compounds for 1 h at 37°C prior to infection.

Subsequently, the cells were infected with the Wuhan-like SARS-CoV-2 strain at a multiplicity of infection (MOI) of 0.3 and incubated for 48 h at 37°C.

#### 2.7.3. Immunofluorescence analysis

For immunofluorescence analysis of SARS-CoV-2-infected cells, the cells were fixed in 4% paraformaldehyde (Sigma) prepared in phosphate-buffered saline (PBS) for 1 h at room temperature (RT). Following fixation, the cells were permeabilized with 0.5% Triton X-100 (Sigma–Aldrich) in PBS for 20 min at RT and then blocked with 10% fetal calf serum (FCS; Anprotec) in PBS for 30 min at RT. The primary antibody, anti-nucleoprotein antibody (Hölzel), was applied at a 1:8000 dilution in PBS containing 10% FCS and incubated overnight at 4°C. Secondary antibody staining was performed using donkey anti-rabbit IgG conjugated to Alexa Fluor 488 (ThermoFisher) at a 1:500 dilution in PBS, alongside 4′,6-diamidino-2-phenylindole (DAPI) staining (Sigma) at a 1:3000 dilution. Incubation with secondary antibodies and DAPI was carried out at RT for 1–2 h. Each step, fixation, permeabilization and antibody staining, was followed by two to three washes with PBS, each lasting 5 min.

Imaging of the fixed and stained cells was conducted using a Celigo Image Cytometer (Nexcelom). Infected cells were quantified using the *ImageJ* software (version 1.53k/Java-1.8.0_172) based on the signal derived from the SARS-CoV-2 nucleoprotein. The percentage of infected cells was calculated by dividing the number of SARS-CoV-2-positive cells by the total number of DAPI-stained nuclei and then multiplying by 100.

## 3. Results

### 3.1. The myricetin binding site in SARS-CoV-2 NSP13

NSP13 of SARS-CoV-2 is a 67 kDa protein with a triangular pyramidal structure comprising five distinct domains: the N-terminal zinc-binding domain (ZBD), the helical stalk domain, the β-barrel 1B domain and two RecA-like domains: RecA1 and RecA2 (Fig. 1 ▸*a*). In addition, a long, unstructured linker connects the domains 1B and RecA1.

The crystal structure of myricetin-bound NSP13 reveals that the inhibitor binds to the surface of the RecA1 domain near its interface with the stalk domain (Fig. 1 ▸*b*). Myricetin interacts with residues from both the RecA1 domain and the linker, which extends across the surface of RecA1. Besides, residual difference density was observed near the ligand, likely originating from an unmodeled symmetry-related loop (residues 204–207 in chain *B*). Although this region lacked sufficient density for reliable modelling, a potential contribution to the binding site cannot be excluded.

The myricetin binding site is situated 32 Å from the nucleotide-binding site and 27 Å from the RNA-binding channel (Supplementary Fig. S2). Notably, this site remains exposed and accessible within the replicase–transcriptase complex (RTC; PDB entry 6xez), highlighting the potential of myricetin to bind and inhibit NSP13 in its functional context (Supplementary Fig. S3).

**Figure 3:**
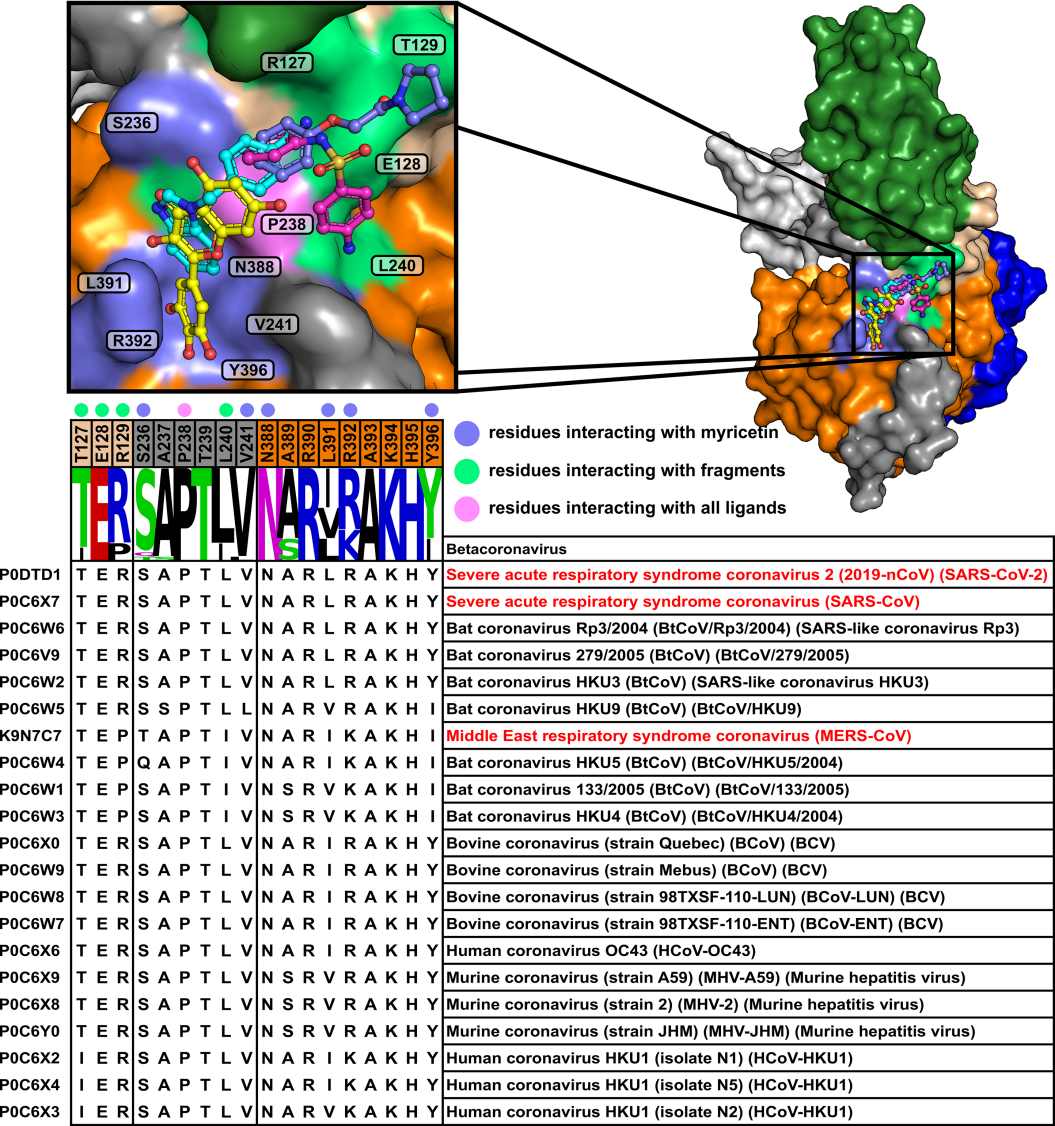
The sequence conservation of myricetin- and fragment-interacting residues in SARS-CoV-2 NSP13. The sequence alignment across 21 beta­coronaviruses highlights residues in SARS-CoV-2 NSP13 that interact with myricetin or the fragments RYM, VVM and VVY (PDB entries 5rme, 5rlc and 5rld, respectively) identified by Newman *et al.* (2021 ▸). Major outbreak-associated viruses are highlighted in red. Residues are visualized in a sequence-logo plot and categorized by their locations within the stalk (wheat), linker (dark grey) and RecA1 (orange) regions. Dots indicate residues interacting with myricetin (violet), fragments (green) or both (pink). The surface representation (top right) shows NSP13 colour-coded as in Fig. 1 ▸, with ligands in ball-and-stick format: myricetin (yellow), RYM (cyan), VVM (magenta) and VVY (blue). An enlarged view of the binding pocket (top left) highlights residues interacting with myricetin and fragments RYM, VVM and VVY.

*In silico* binding-affinity assessments revealed that the pyrogallol moiety of myricetin, comprising ring B and its substituents, significantly contributes to NSP13 binding (Fig. 2 ▸). Interaction analysis (Fig. 1 ▸) showed that the pyrogallol moiety forms hydrogen bonds via its 5′-OH and 4′-OH groups to Tyr396, and its 5′-OH group additionally interacts with Arg392 and the main chains of Asn388 and Leu391. Further, carbon–π interactions were also identified between ring B and Val241, while Arg392 engages in both cation–π and donor–π interactions with ring B. The trihydroxychromone moiety, encompassing rings A and C and their substituents, contributes to binding through hydrogen bonds, with its 3-OH group interacting with Arg392 and a water molecule, which also engages the 4-OH group, while the 5-OH group forms a hydrogen bond to the main chain of Ser236. Additionally, both ring A and ring C exhibit carbon–π interactions with Pro238. Sequence-conservation analysis of NSP13 from SARS-CoV-2 and related betacoronaviruses, including SARS-CoV-1 and MERS-CoV, revealed that these key interacting residues are highly conserved (Fig. 3 ▸).

Based on these findings, it was hypothesized that myricetin forms a conserved interaction network with NSP13. To test this hypothesis, a structural alignment was performed, revealing that NSP13 structures from SARS-CoV-1 (PDB entry 6jyt) and MERS-CoV (PDB entry 5wwp) have nearly identical residues in the same spatial arrangement compared with the myricetin binding site in SARS-CoV-2 (Supplementary Fig. S4). The only exceptions are arginine (Arg392) and serine (Ser236), which are replaced by lysine and threonine, respectively, in MERS-CoV NSP13. This observation aligns with the sequence and structural conservation observed in SARS-CoV-2 NSP13 when compared with SARS-CoV-1 (99.8% sequence identity, C^α^ r.m.s.d. 1.17 Å) and MERS-CoV (71% sequence identity, C^α^ r.m.s.d. 0.95 Å). These findings underscore the high conservation of this binding site in NSP13.

### 3.2. Fragments in the vicinity of the myricetin binding site

**Figure 4:**
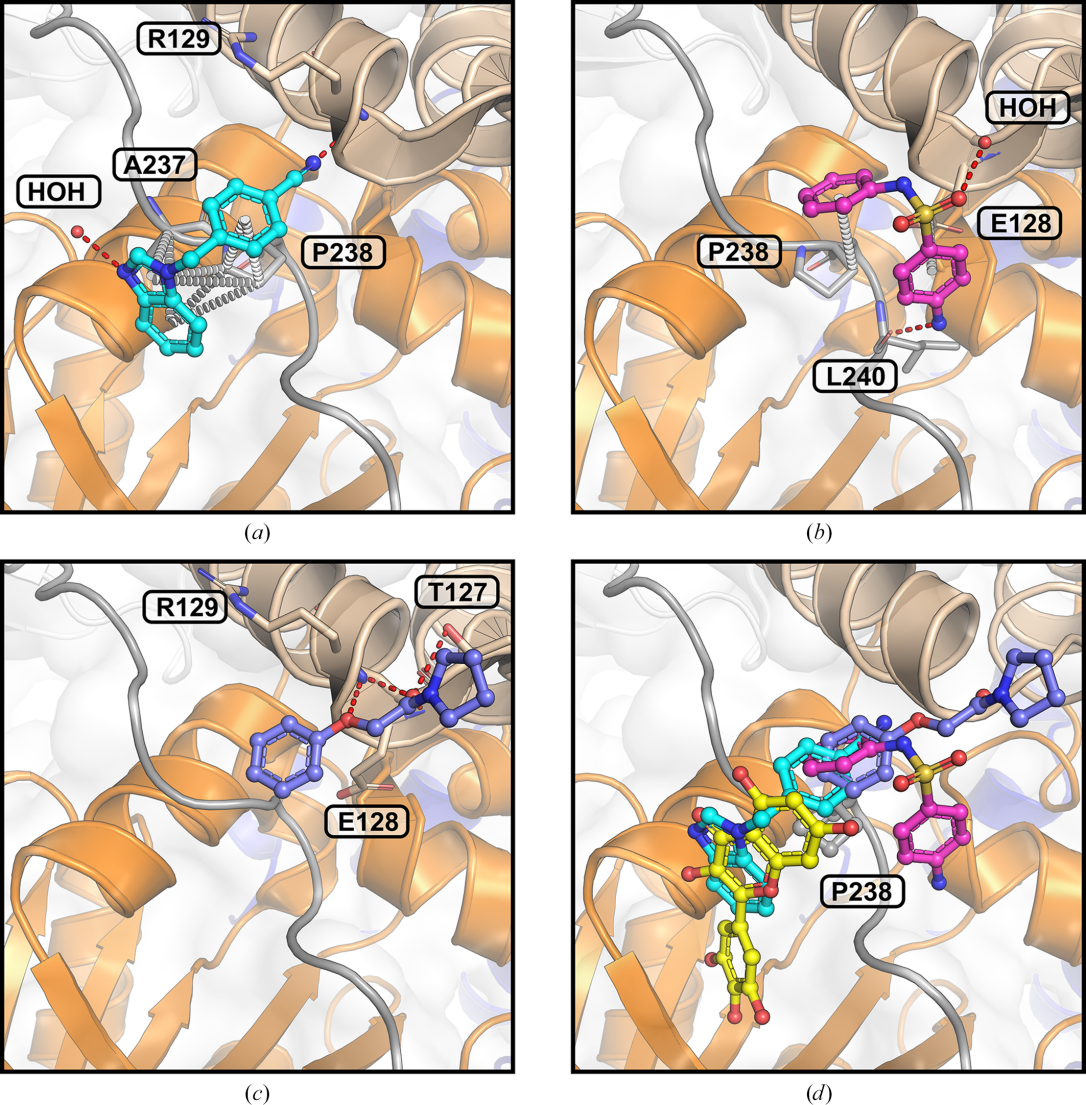
Fragments in the vicinity of the myricetin binding site in SARS-CoV-2 NSP13. NSP13 is shown in cartoon representation as in Fig. 1 ▸, with ligands depicted in ball-and-stick format. Fragments identified by Newman *et al.* (2021 ▸) that overlap with or are adjacent to the myricetin binding site are illustrated: (*a*) fragment RYM (cyan; PDB entry 5rme), (*b*) fragment VVM (magenta; PDB entry 5rlc) and (*c*) fragment VVY (blue; PDB entry 5rld). Interacting residues are shown as sticks and water molecules are represented as spheres; each are labelled. Hydrogen bonds (red dashed lines) and carbon–π interactions (white dashed lines) are indicated. (*d*) Myricetin (yellow) and the fragments collectively form an extensive interaction network centred around Pro238, linking the RecA1 and stalk domains of NSP13.

The myricetin binding site was analysed in the context of the crystallographic fragment screen conducted by Newman *et  al.* (2021 ▸). Three fragments, RYM (PDB entry 5rme), VVM (PDB entry 5rlc) and VVY (PDB entry 5rld), were identified as either overlapping with or adjacent to the myricetin binding site, as shown in Fig. 4 ▸. The interactions between these fragments and NSP13, along with the conservation of their interacting residues, were systematically examined.

Fragment RYM overlaps with myricetin, interacting with Pro238 and Ala237 through carbon–π interactions. It engages the same water molecule as myricetin via a hydrogen bond and forms an additional hydrogen bond to the main chain of Arg129. Fragment VVM, which overlaps with RYM, establishes carbon–π interactions with Pro238 and Glu128 and forms hydrogen bonds to a water molecule and the main chain of Leu240. Fragment VVY, overlapping with VVM, forms hydrogen bonds to the side chain of Thr127 and the main chains of Glu128 and Arg129. The residues interacting with these fragments are highly conserved across NSP13 from SARS-CoV-2 and related betacoronaviruses (Fig. 3 ▸).

In conjunction with myricetin, these fragments form an extensive interaction network centred around Pro238, highlighting its potential as a key scaffold for inhibitor development (Fig. 4 ▸*d*). The structural insights from these fragment-bound structures were incorporated into the virtual screening campaign, refining the query for the similarity search in *ROCS*.

### 3.3. *In silico*-based identification of inhibitors against SARS-CoV-2 NSP13

**Figure 5:**
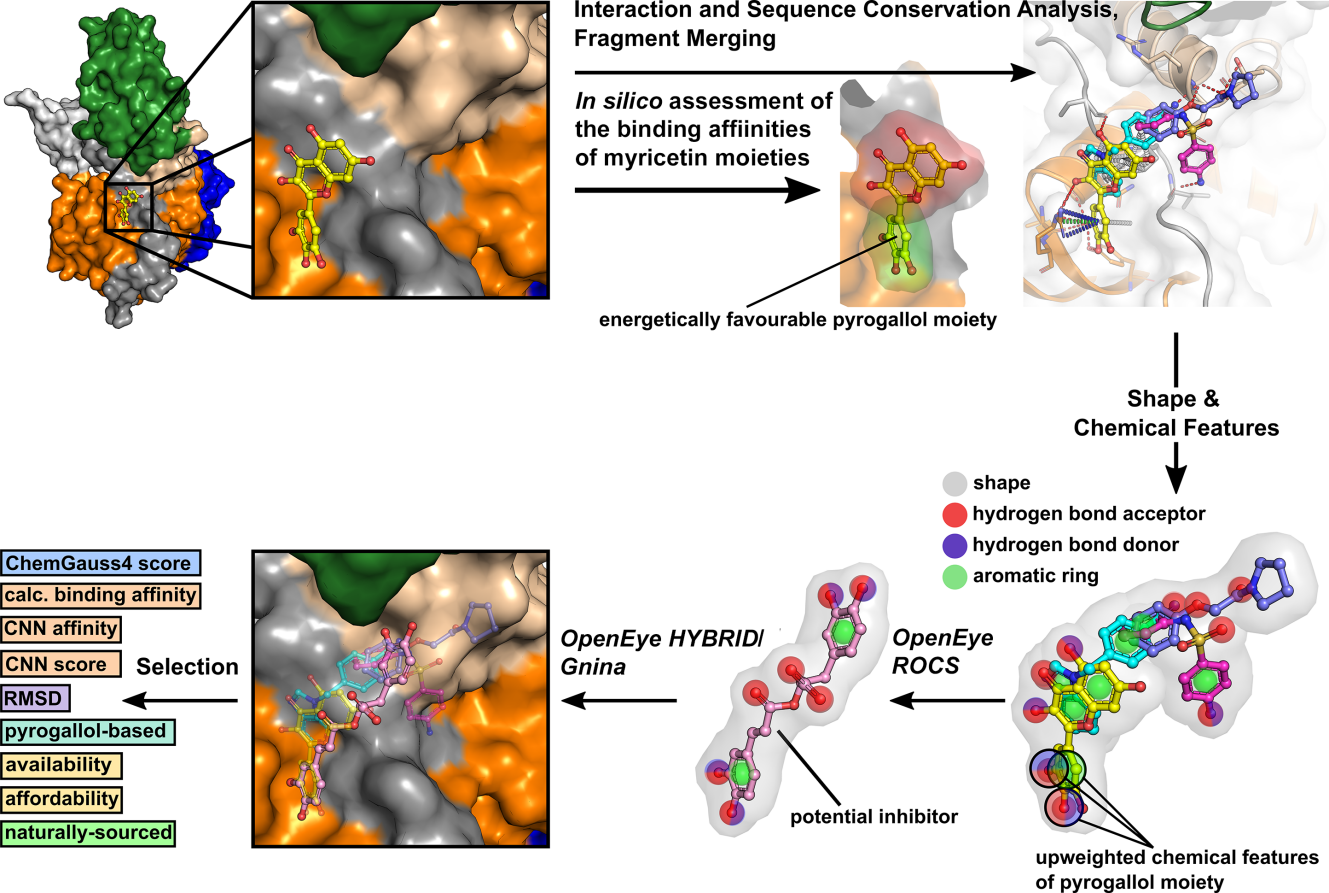
The workflow of the virtual screening campaign to identify inhibitors of SARS-CoV-2 NSP13. The process begins (top left) with an analysis of the myricetin binding site on NSP13. The myricetin conformer served as the foundation for the *ROCS* query, which was refined through interaction and sequence-conservation analyses and extended through incorporating fragments identified by Newman *et al.* (2021 ▸). The *in silico* assessment of the binding affinities of the myricetin moieties identified the pyrogallol moiety (green) as energetically favourable for binding to NSP13 compared with the trihydroxychromone moiety (red). These structural and computational insights guided the design of a *ROCS* query that combined the shapes (grey) of myricetin and the fragments while incorporating conserved chemical features such as hydrogen-bond donors (blue), acceptors (red) and aromatic rings (green). The energetically favourable features of the pyrogallol moiety were upweighted to prioritize pyrogallol-based compounds. *ROCS* was then employed to search for compounds matching the shape and chemical features of the query, with the results further refined through docking using *HYBRID* and *Gnina*. Promising candidates were selected based on ChemGauss4 scores, binding affinities, CNN scores, r.m.s.d. values, the presence of pyrogallol-like moieties and the availability and affordability of the compounds, which are naturally sourced.

Protein–inhibitor complex structures provide a robust foundation for both ligand-based and structure-based *in silico* drug discovery. Myricetin, a flavonoid with notable inhibitory activity in both enzymatic and cell-based assays, represents a promising scaffold for inhibitor development. To identify novel inhibitors targeting the SARS-CoV-2 helicase, structural information from the myricetin-bound NSP13 complex, combined with fragment-bound structures (Newman *et al.*, 2021 ▸), was used in the virtual screening strategy outlined by Garbers *et al.* (2024 ▸). This approach integrates ligand-based and structure-based methods, as described in Section 2.5 and illustrated in Fig. 5 ▸.

Insights from structural analysis of the myricetin binding site in NSP13, including its conformer binding, interactions, protein sequence conservation and *in silico* assessment of the binding affinities of its moieties (Figs. 1 ▸, 2 ▸ and 3 ▸), were incorporated into the *ROCS* query (Fig. 5 ▸). Additionally, structural data from the crystallographic fragment screen by Newman *et al.* (2021 ▸) expanded the query to include conserved regions beyond the myricetin binding site (Fig. 4 ▸). The final *ROCS* query combined the molecular shapes of myricetin and three additional fragments (RYM, VVM and VVY), incorporating chemical features reflecting their conserved interactions with NSP13. The pyrogallol moiety, which is predicted to be critical for myricetin binding, was prioritized by applying additional weights (Supplementary Fig. S6). Ligand-based screening with *ROCS* was complemented by molecular-docking tools, with *HYBRID* re-evaluating the top *ROCS* hits in the context of the NSP13 receptor and *Gnina* cross-validating the *HYBRID* results. This combined approach identified compounds that matched the shape and chemical features of the query while adopting favourable docking poses within the binding site of NSP13.

Promising inhibitors were selected based on high scores and calculated affinities from both docking programs, as well as minimal r.m.s.d. between poses docked by *HYBRID* and *Gnina*. The selected hits were visually inspected in *PyMOL*, focusing on their interactions with NSP13 and the presence of a pyrogallol-like moiety prioritized during *ROCS*. Inspired by the antiviral potential of the flavonoid myricetin and the historical significance of natural compounds to drug discovery, the evaluation of the *ROCS* results prioritized affordable and readily available compounds, which are naturally sourced.

**Figure 6:**
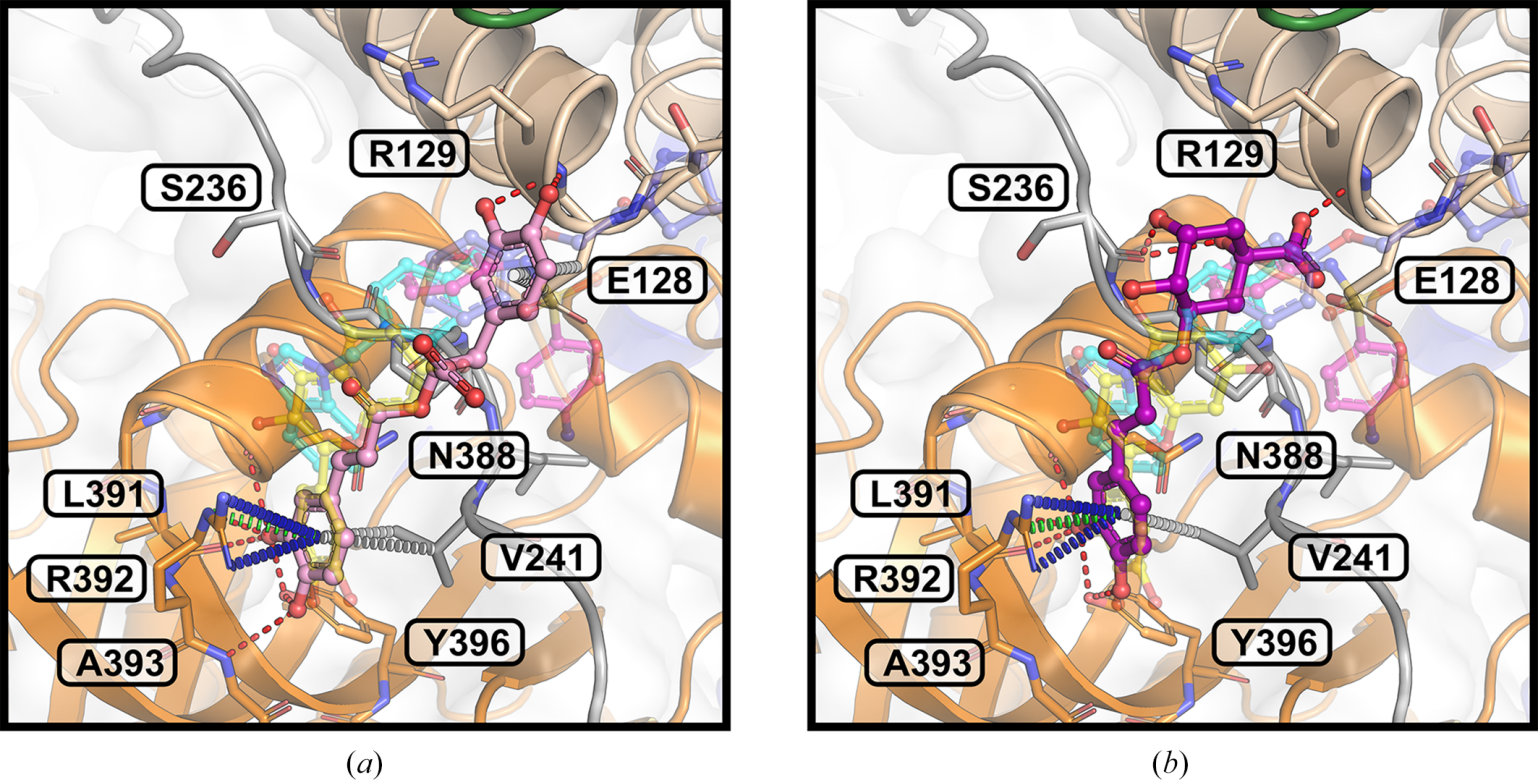
Docking results for rosmarinic acid and chlorogenic acid within the binding site of SARS-CoV-2 NSP13. Rosmarinic acid (*a*) and chlorogenic acid (*b*) are shown in ball-and-stick format in pink and violet, respectively, with NSP13 in cartoon representation coloured as in Fig. 1 ▸. Interacting residues are depicted as sticks and labelled, indicating hydrogen bonds (red dashed lines), carbon–π interactions (white dashed lines), cation–π interaction (green dashed lines) and donor–π interactions (blue dashed lines). Myricetin and fragments are displayed transparently in the same colours as in Figs. 1 ▸ and 2 ▸.

Rosmarinic acid and chlorogenic acid emerged as the most promising potential inhibitors of SARS-CoV-2 NSP13. Both natural compounds are derivatives of caffeic acid and contain a pyrocatechol moiety, characterized by a benzene ring with two hydroxyl groups in the *ortho* position, resembling the pyrogallol moiety of myricetin. Their chemical structures are shown in Table 3 ▸. Interaction analysis of the docked conformers revealed binding patterns similar to those observed in the myricetin-bound and fragment-bound crystal structures (Fig. 6 ▸). Rosmarinic acid formed hydrogen bonds to Arg129, Asn388, Leu391, Arg392, Ala393 and Tyr396, as well as carbon–π interactions with Glu128 and Val241. Additionally, it established cation and donor–π interactions with Arg392. Chlorogenic acid displayed a comparable interaction profile, forming hydrogen bonds to Arg129, Ser236, Asn388, Leu391, Arg392 and Tyr396, along with carbon–π interactions with Val241. It also exhibited cation and donor–π interactions with Arg392. These findings underscore the potential of rosmarinic acid and chlorogenic acid as natural product-based inhibitors of SARS-CoV-2 NSP13.

### 3.4. Derivatives of caffeic acid inhibit SARS-CoV-2 NSP13

Rosmarinic acid and chlorogenic acid were identified *in silico* as potential inhibitors of SARS-CoV-2 NSP13 and were subsequently validated *in vitro*. Both compounds, along with myricetin, were tested in ATPase and unwinding assays. Myricetin inhibited both activities of NSP13, consistent with previous studies (Corona *et al.*, 2022 ▸; Kuzikov *et al.*, 2024 ▸; Inniss *et al.*, 2024 ▸). In contrast, rosmarinic acid and chlorogenic acid inhibited only the unwinding activity of NSP13 (Table 5 ▸, Supplementary Fig. S7). In cell-based infection assays, rosmarinic acid demonstrated an IC_50_ of 59.31 µ*M*, whereas chlorogenic acid exhibited no significant inhibition (Supplementary Fig. S8).

## 4. Discussion

### 4.1. Identification of a novel allosteric site in NSP13

In order to advance antiviral therapeutic development against SARS-CoV-2, the crystal structure of myricetin-bound NSP13 was determined, providing detailed insights into the myricetin binding site, which is distant from the active sites (Fig. 1 ▸, Supplementary Fig. S2). Prior to this study, ligand-bound NSP13 structures were limited to nucleotide-bound or RNA-bound forms and a crystallographic fragment screen, leaving significant opportunities for structure-based drug design unexplored. The myricetin-bound structure addresses this gap by identifying a novel allosteric site with characteristics favourable for drug development. This site remains accessible to small molecules within the replicase–transcriptase complex (RTC), enabling targeting of NSP13 during SARS-CoV-2 replication (Supplementary Fig. S3). Its high sequence conservation among betacoronaviruses further emphasizes its potential as a pan-coronavirus therapeutic target (Fig. 3 ▸). The myricetin binding site offers an attractive alternative to the ATP-binding site or the RNA-binding channel, which are more prone to off-target effects, as exemplified by their structural similarity to the human nonsense-mediated mRNA decay factor UPF1 (Jia *et al.*, 2019 ▸; Newman *et al.*, 2021 ▸). Key insights from the myricetin-bound NSP13 structure, including the energetically favourable pyrogallol moiety, interaction patterns and sequence conservation (Figs. 1 ▸, 2 ▸ and 3 ▸), guided the virtual screening campaign illustrated in Fig. 5 ▸. Data from the crystallographic fragment screen (Newman *et al.*, 2021 ▸) expanded the search for ligands beyond the myricetin binding site (Fig. 4 ▸), leading to the identification of rosmarinic acid and chlorogenic acid (Fig. 6 ▸). Both natural products were biochemically validated to inhibit the unwinding activity of NSP13 more potently than myricetin (Table 5 ▸, Supplementary Fig. S7). These findings highlight the structural and functional importance of the myricetin-bound NSP13 complex, providing insights into its inhibition mechanism and supporting the development of novel, naturally derived inhibitors against SARS-CoV-2.

### 4.2. Myricetin as a dual-function inhibitor of NSP13

**Figure 7:**
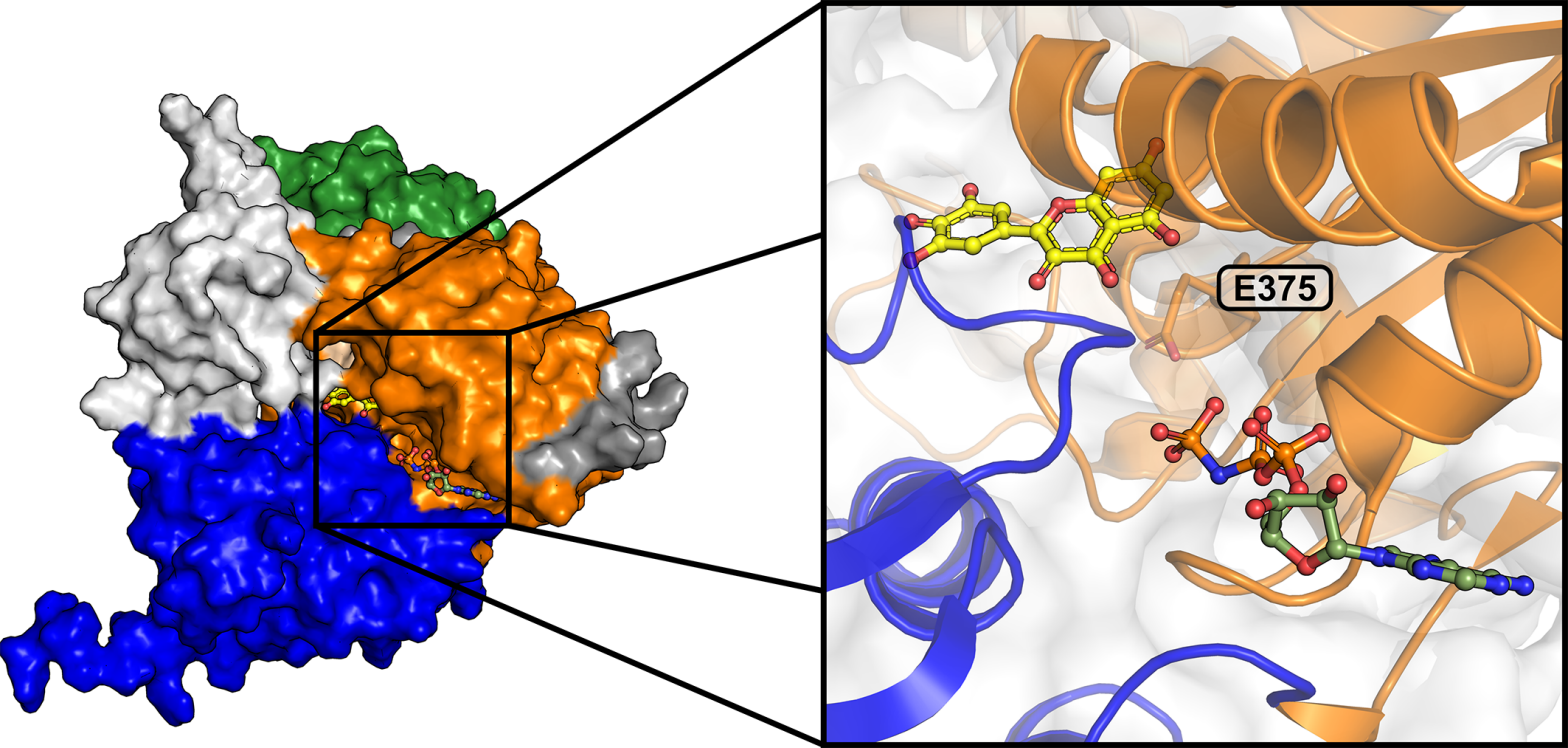
Predicted binding mode of myricetin at the nucleotide-binding cleft of SARS-CoV-2 NSP13. The NSP13 structure in complex with myricetin, as predicted by *RoseTTAFold All-Atom*, is shown in surface representation, highlighting the nucleotide-binding cleft formed by the RecA1 (orange) and RecA2 (blue) domains. Myricetin (yellow) and AMP-PNP from PDB entry 7nn0 are depicted in ball-and-stick format, indicating the location of the cleft. The inset provides a detailed view of myricetin positioned near the catalytic residue Glu375.

The inhibition of SARS-CoV-2 NSP13 by myricetin has been demonstrated in multiple *in vitro* studies, although the results have varied, as summarized in Table 6 ▸. Early studies reported that myricetin inhibited only the ATPase activity (Yu *et al.*, 2012 ▸), whereas more recent investigations, including this one, demonstrated inhibition of both the ATPase and unwinding activities (Table 5 ▸; Corona *et al.*, 2022 ▸; Kuzikov *et al.*, 2024 ▸; Inniss *et al.*, 2024 ▸). The virtual screening based on the myricetin-bound structure identified rosmarinic acid and chlorogenic acid as potential inhibitors of NSP13. Both were designed to specifically target the myricetin binding site reported here, and their binding modes were predicted by docking (Fig. 6 ▸), as soaking and co-crystallization experiments were unsuccessful. Biochemical analyses revealed their ability to inhibit unwinding activity *in vitro* (Table 5 ▸, Supplementary Fig. S7), supporting the hypothesis that the reported myricetin binding site regulates this function of NSP13. Interestingly, the observed inhibition of ATPase activity suggests the presence of a secondary myricetin binding site, likely distinct from that described in the structure reported here. Using *RoseTTAFold All-Atom* (Krishna *et al.*, 2024 ▸), a deep-learning model capable of predicting protein–small molecule complex structures, myricetin was shown to bind near the nucleotide-binding cleft of NSP13 (Fig. 7 ▸). Notably, a similar binding mode was observed in the bacterial chaperone protein DnaK, where myricetin bound ‘above’ the nucleotide-binding cleft, altering its activity (Chang *et al.*, 2011 ▸). To explore whether the second binding site is specific to myricetin, we also evaluated rosmarinic acid and chlorogenic acid using *RoseTTAFoldAll-Atom*. Neither compound was predicted to bind at the proposed site near the nucleotide-binding cleft. Instead, both were predicted to bind at the 5′ end of the RNA-binding channel, similar to the binding mode observed for MOPS (Supplementary Fig. S1). These findings are consistent with their lack of ATPase inhibition and further support the specificity of myricetin for the secondary site near the nucleotide-binding cleft.

### 4.3. Allosteric regulation of NSP13 by myricetin

**Figure 8:**
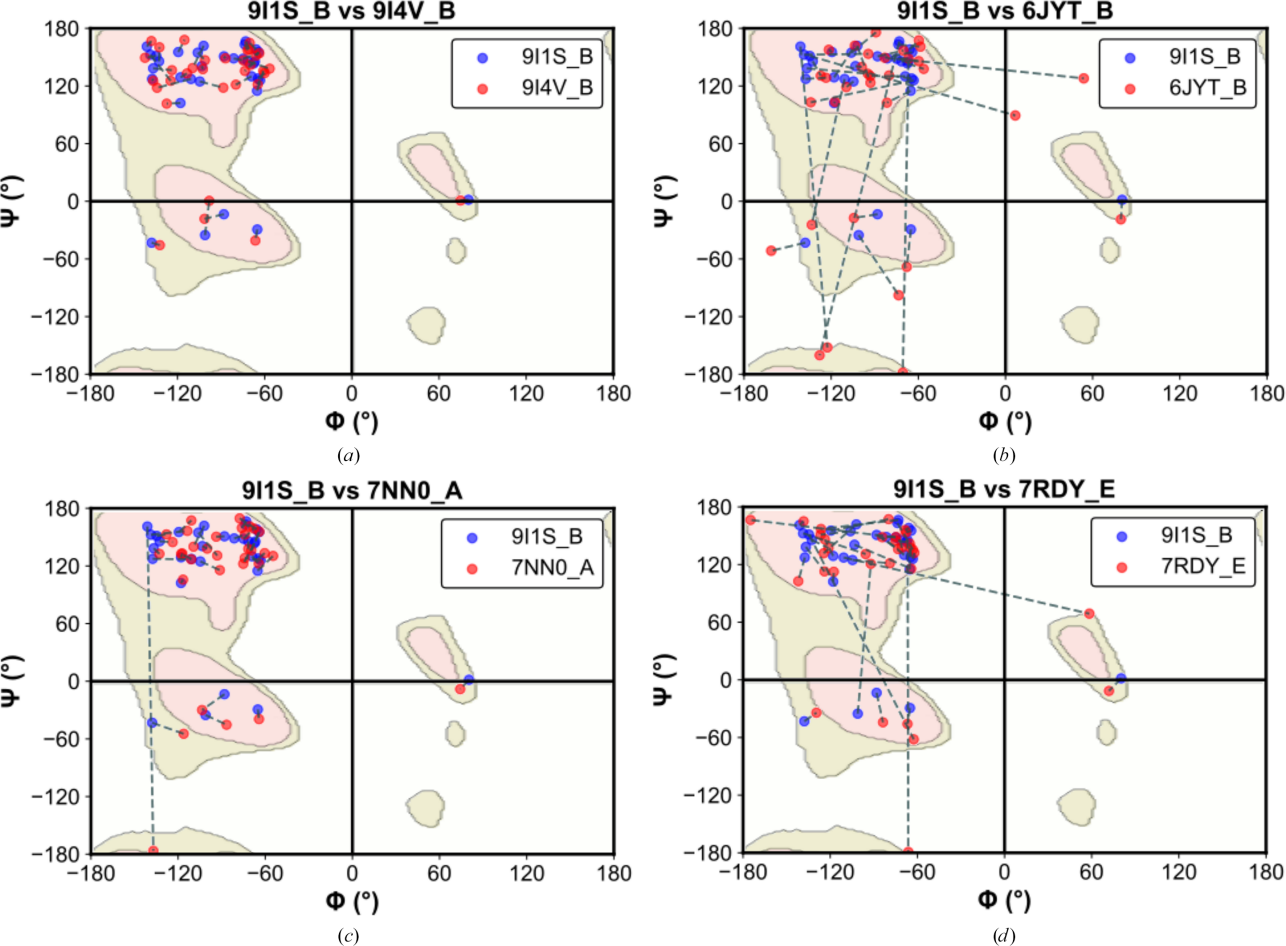
Kleywegt plots comparing the backbone dihedral angles (Φ, Ψ) for the linker region connecting the 1B and RecA1 domains across structures of NSP13. The plots compare the myricetin-bound SARS-CoV-2 NSP13 crystal structure (PDB entry 9i1s, chain *B*) with the crystal structures of (*a*) unliganded SARS-CoV-2 NSP13 (PDB entry 9i4v, chain *B*), (*b*) SARS-CoV-1 NSP13 (PDB entry 6jyt, chain *B*) and (*c*) AMP-PNP-bound SARS-CoV-2 NSP13 (PDB entry 7nn0, chain *A*), as well as (*d*) the RNA-bound cryo-EM structure of SARS-CoV-2 NSP13 (PDB entry 7rdy, chain *E*). Dashed lines connect corresponding residues in each model, highlighting positional shifts, while contour lines indicate favoured and allowed regions in the multiple-model Ramachandran plot.

In the structure reported here, myricetin binds to the RecA1 domain and the linker connecting the 1B and RecA1 domains (Fig. 1 ▸), positioned distant from the ATP- and RNA-binding sites of NSP13 (Supplementary Fig. S3). This binding site is predicted to influence the unwinding activity of NSP13, as previously discussed. To investigate the underlying inhibition mechanism, the myricetin-bound structure (PDB entry 9i1s) was compared with an unliganded NSP13 structure crystallized under identical conditions (PDB entry 9i4v). No significant structural differences were detected, as reflected by a C^α^ r.m.s.d. of 0.20 Å. Moreover, an analysis of Φ and Ψ backbone angles, visualized using the Kleywegt plot (Kleywegt & Jones, 1996 ▸), indicated no notable local alterations (Fig. 8 ▸). The myricetin-bound structure was obtained through crystal soaking after co-crystallization attempts proved unsuccessful. However, crystal packing may hinder the observation of ligand-induced conformational changes when the soaking method is used (Ehrmann *et al.*, 2017 ▸; Wienen-Schmidt *et al.*, 2021 ▸). Further comparisons with the SARS-CoV-1 NSP13 crystal structure (PDB entry 6jyt), the SARS-CoV-2 NSP13 crystal structure in complex with AMP-PNP (PDB entry 7nn0) and an RNA-bound cryo-EM structure (PDB entry 7rdy) revealed significant movements in the linker region connecting the 1B and RecA1 domains (Fig. 8 ▸). Interdomain linkers are known to transmit conformational changes between sites in response to ligand binding (Ma *et al.*, 2011 ▸). These findings suggest that myricetin binding could alter the conformational landscape of this linker region, potentially reducing its flexibility and allosterically inhibiting the unwinding activity of NSP13. To further support this mechanistic model, future studies involving site-directed mutagenesis of residues within the proposed binding site would be of interest to confirm the functional role of this region in NSP13.

### 4.4. Myricetin and caffeic acid derivatives as inhibitors of SARS-CoV-2

Myricetin has been shown to interact with multiple SARS-CoV-2 proteins beyond NSP13, including the spike protein (*K*_d_ = 2.67 µ*M*), main protease (NSP5; IC_50_ = 0.63 µ*M*) and RNA-dependent RNA polymerase (NSP12; IC_50_ = 0.86 µ*M*) (Su *et al.*, 2021 ▸; Pan *et al.*, 2023 ▸; Kuzikov *et al.*, 2024 ▸). Beyond the myricetin–NSP13 structure reported here, myricetin has also been crystallized in complex with the main protease NSP5, where it covalently binds to the catalytic cysteine, identifying the pyrogallol moiety as an electrophilic warhead (Su *et al.*, 2021 ▸). Furthermore, cell-based studies have reported the inhibition of SARS-CoV-2 replication by myricetin, with IC_50_ values ranging between 8.00 and 55.18 µ*M* (Su *et al.*, 2021 ▸; Pan *et al.*, 2023 ▸). These findings highlight myricetin, a naturally occurring flavonoid, as a promising yet improvable inhibitor against SARS-CoV-2. Inspired by the significant contributions of natural products, the virtual screen conducted here aimed to identify new, more efficient natural inhibitors of NSP13. Rosmarinic acid and chlorogenic acid emerged as potent inhibitors of the unwinding activity of NSP13 (Table 5 ▸, Supplementary Fig. S7). Interestingly, both have also been reported to inhibit other SARS-CoV-2 proteins. Rosmarinic acid inhibits NSP5 with an IC_50_ of 2.18 µ*M*, binding non­covalently to its active site, as revealed by structural characterization (Li *et al.*, 2024 ▸). Likewise, chlorogenic acid has been shown to inhibit the papain-like protease (NSP3) and prevent the interaction of the spike protein with the ACE2 receptor (Abomughaid *et al.*, 2022 ▸; Hsieh *et al.*, 2024 ▸). Despite promising results in enzymatic assays, the cell-based infection assays showed no improvement over myricetin, as rosmarinic acid exhibited an IC_50_ of 59.31 µ*M*, while chlorogenic acid showed no significant inhibition (Supplementary Fig. S8). Neither caffeic acid derivative demonstrated sufficient efficacy in cell-based assays to be considered a potential lead compound. Both rosmarinic acid and chlorogenic acid are reported to have limited solubility, chemical stability and cell permeability, which may reduce their effective concentrations in cell-based systems (Chaitanya *et al.*, 2022 ▸; Trivedi & Puranik, 2023 ▸). Moreover, reduced cell numbers at 200 µ*M* rosmarinic acid (Supplementary Fig. S8) suggest cytotoxicity at higher concentrations, which may have contributed to the apparent antiviral effect observed in the infection assay. To address this issue, alternative strategies should be explored. For instance, Su *et al.* (2021 ▸) significantly enhanced the bio­availability of myricetin through small chemical modifications based on the structure of its complex with NSP5. This approach demonstrated the potential for myricetin to be developed as an orally administrable drug against SARS-CoV-2.

### 4.5. Rational design of natural product-based inhibitors using structural insights

The myricetin-bound crystal structure of NSP13 reveals an allosteric binding site with substantial therapeutic potential. Insights from this protein–inhibitor complex guided the virtual screening campaign, leading to the identification of rosmarinic acid and chlorogenic acid as novel natural inhibitors against NSP13. These findings highlight the critical role of structural biology in antiviral drug development. Although bioavailability remains a significant challenge, the structural insights presented here could contribute to the rational design of more potent and bioavailable inhibitors targeting NSP13 in SARS-CoV-2 and other coronaviruses with pandemic potential.

## Supplementary Material

PDB reference: SARS-CoV-2 helicase NSP13, 9i4v

PDB reference: complex with myricetin, 9i1s

Supplementary Figures. DOI: 10.1107/S2059798325004498/ud5057sup1.pdf

## Figures and Tables

**Table 1:** Crystallization

Method	Vapour diffusion
Plate type	Sitting drop
Temperature (K)	293
Protein concentration (mg ml^−1^)	5
Buffer composition of protein solution	12.5 m*M* HEPES pH 7.5, 125 m*M* NaCl, 0.125 m*M* TCEP–HCl
Composition of reservoir solution	16% ethylene glycol, 8% PEG 8000, 0.05 *M* HEPES, 0.05 *M* MOPS, 0.03 *M* sodium nitrate, 0.03 *M* sodium phosphate, 0.03 *M* ammonium sulfate, 9% MPD†
Volume and ratio of drop	0.5 µl, 1:1 ratio
Volume of reservoir (µl)	40
Composition of cryoprotectant	20% ethylene glycol, 10% PEG 8000, 0.05 *M* HEPES, 0.05 *M* MOPS, 0.03 *M* sodium nitrate, 0.03 *M* sodium phosphate, 0.03 *M* ammonium sulfate, 9% MPD†
Seeding type	Microseeding
Volume of seeds (µl)	0.033

† MPD was added to modify the conditions from Newman *et al.* (2021 ▸).

**Table 2:** Data collection and processing Values in parentheses are for the outer shell.

Structure	SARS-CoV-2 NSP13	Myricetin-bound NSP13
PDB code	9i4v	9i1s
Diffraction source	P13, PETRA III, DESY	P13, PETRA III, DESY
Wavelength (Å)	0.97625	0.97626
Temperature (K)	100	100
Detector	EIGER 16M	EIGER 16M
Crystal-to-detector distance (mm)	252	252
Rotation range per image (°)	0.1	0.1
Total rotation range (°)	500	500
Exposure time per image (s)	0.008	0.008
Space group	*P*1	*P*1
*a*, *b*, *c* (Å)	59.305, 70.687, 86.225	59.327, 70.632, 86.149
α, β, γ (°)	103.704, 95.236, 112.116	103.106, 96.097, 112.149
Mosaicity (°)	0.2	0.2
Resolution range (Å)	82.070–2.325 (2.365–2.325)	62.615–2.088 (2.124 –2.088)
Total No. of reflections	186790 (10131)	255978 (8172)
No. of unique reflections	49334 (2573)	67228 (3108)
Completeness (%)	92.8 (95.2)	91.8 (85.2)
Multiplicity	3.8 (3.9)	3.8 (2.6)
〈*I*/σ(*I*)〉	6.7 (2.2)	11.9 (2.4)
*R*_meas_	0.068 (0.504)	0.077 (0.812)
Overall *B* factor from Wilson plot (Å^2^)	47.3582	43.7657

**Table 3:** Molecules

Name	Molecule	IUPAC name	CAS ID
Myricetin	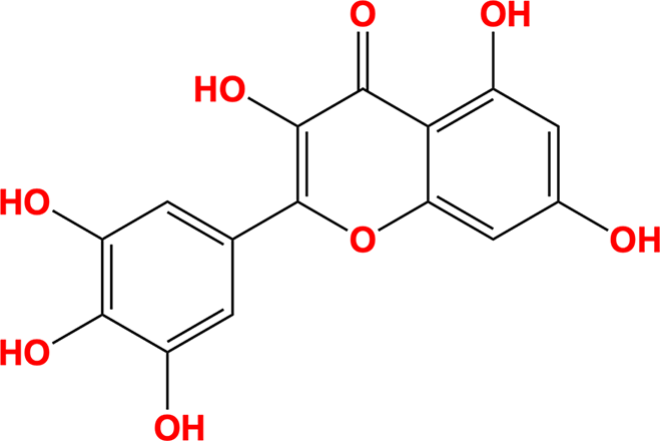	3,5,7-Trihydroxy-2-(3,4,5-trihydroxyphenyl)chromen-4-one	529-44-2
Pyrogallol	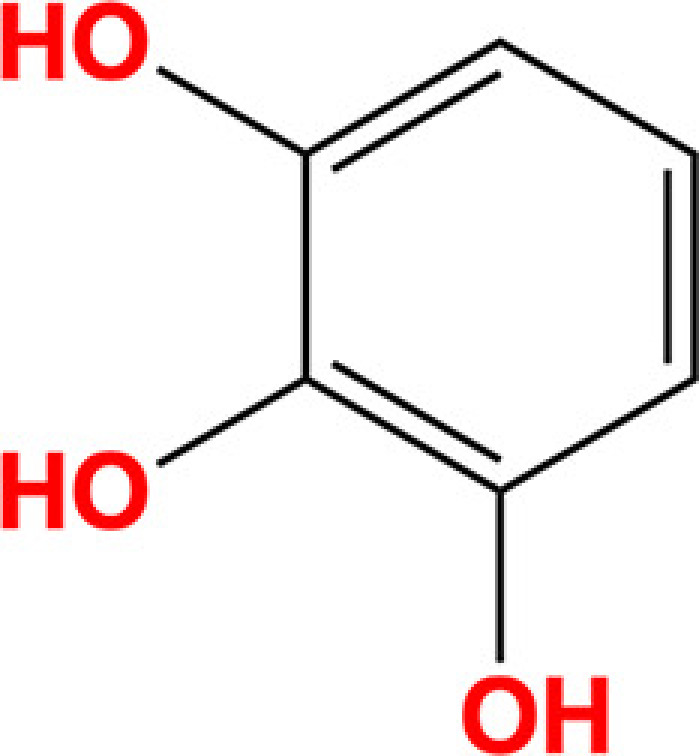	Benzene-1,2,3-triol	87-66-1
Trihydroxychromone	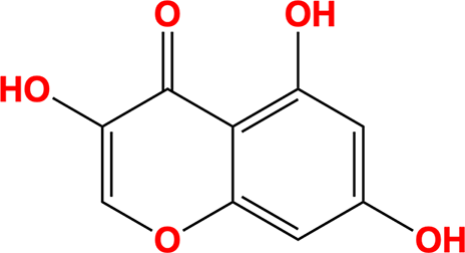	3,5,7-Trihydroxychromen-4-one	31721-95-6
RYM (from PDB entry 5rme)†	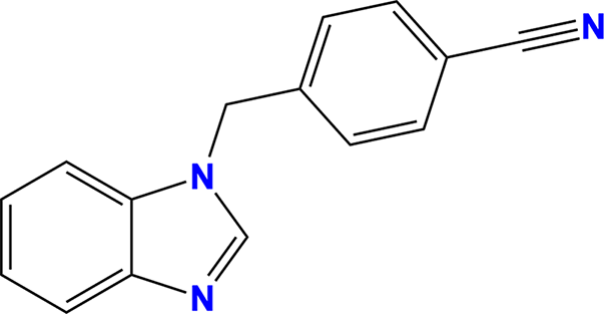	4-(Benzimidazol-1-ylmethyl)benzonitrile	118001-91-5
VVM (from PDB entry 5rlc)†	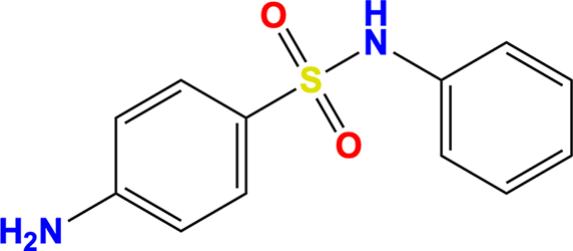	4-Amino-*N*-phenylbenzenesulfonamide	127-77-5
VVY (from PDB entry 5rld)†	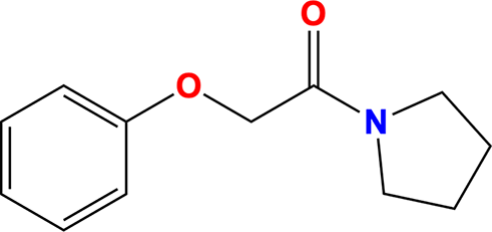	4-(Benzimidazol-1-ylmethyl)benzonitrile	112283-41-7
Rosmarinic acid	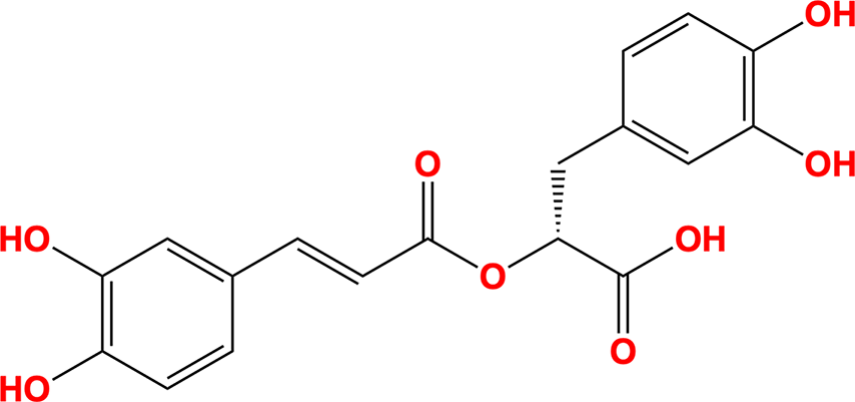	(2*R*)-3-(3,4-Dihydroxyphenyl)-2-[(*E*)-3-(3,4-dihydroxy­phenyl)prop-2-enoyl]oxypropanoic acid	20283-92-5
Chlorogenic acid	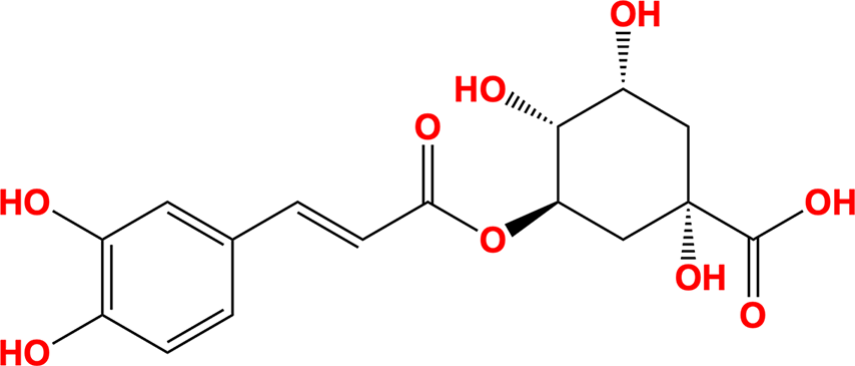	(1*S*,3*R*,4*R*,5*R*)-3-[(*E*)-3-(3,4-Dihydroxyphenyl)prop-2-enoyl]oxy-1,4,5-trihydroxycyclohexane-1-carboxylic acid	202650-88-2
MOPS	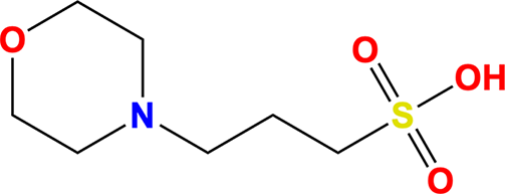	3-Morpholin-4-ylpropane-1-sulfonic acid	1132-61-2

† Fragments identified as binding to SARS-CoV-2 NSP13 by Newman *et al.* (2021 ▸).

**Table 4:** Structure solution and refinement Values in parentheses are for the outer shell.

Structure	SARS-CoV-2 NSP13	Myricetin-bound NSP13
PDB code	9i4v	9i1s
Resolution range (Å)	45.60–2.33 (2.37–2.33)	35.63–2.09 (2.12–2.09)
No. of reflections		
Working set	49319 (2707)	67055 (2423)
Test set	2509 (141)	3349 (145)
Final *R*_cryst_	0.2154 (0.3036)	0.2161 (0.3685)
Final *R*_free_	0.2542 (0.3559)	0.2412 (0.4148)
No. of non-H atoms		
Total	9066	9222
Protein	8911	8917
Ligand	52	85
Water	103	230
Protein residues	1155	1156
R.m.s. deviations		
Bond lengths (Å)	0.004	0.003
Angles (°)	0.914	0.77
Average *B* factors (Å^2^)		
Overall	73.30	65.77
Protein	73.58	66.17
Ligand	60.10	63.06
Water	56.09	50.86
Ramachandran plot		
Most favoured (%)	95.87	95.18
Allowed (%)	3.95	4.74
Outliers (%)	0.18	0.09
Rotamer outliers (%)	1.12	2.24
Clashscore	6.09	3.00

**Table 5:** Results of the ATPase and unwinding assay

Compound	IC_50_, ATPase activity (µ*M*)	IC_50_, unwinding activity (µ*M*)
Myricetin	0.72	7.91
Rosmarinic acid	No inhibition	3.83
Chlorogenic acid	No inhibition	0.87

**Table 6:** Reported inhibitory effects of myricetin on the ATPase and unwinding activities of SARS-CoV-2 NSP13

	Inhibition of ATPase activity	IC_50_, unwinding activity (µ*M*)
Yu *et al.* (2012 ▸)	90% at 10 µ*M*	No inhibition
Corona *et al.* (2022 ▸)	13% at 30 µ*M*	0.41
Inniss *et al.* (2024 ▸)	Not measured	11.0
Kuzikov *et al.* (2024 ▸)	62% at 2 µ*M*	4.14
