# Supplementary material for: Myricetin-bound crystal structure of the SARS-CoV-2 helicase NSP13 facilitates the discovery of novel natural inhibitors
Source: Acta Crystallogr D Struct Biol. 2025 May 27;81(Pt 6):310–26. doi: 10.1107/S2059798325004498 (PMC12128885; doi:10.1107/S2059798325004498)
Supplement: Supplementary file 1 [file d-81-00310-sup1.pdf]

STRUCTURAL
BIOLOGY

Volume 81 (2025)

Supporting information for article:

Myricetin-bound crystal structure of the SARS-CoV-2 helicase NSP13 facilitates the discovery of novel natural inhibitors

Patrick Kloskowski, Piotr Neumann, Priya Kumar, Annette Berndt, Matthias Dobbelstein and Ralf Ficner

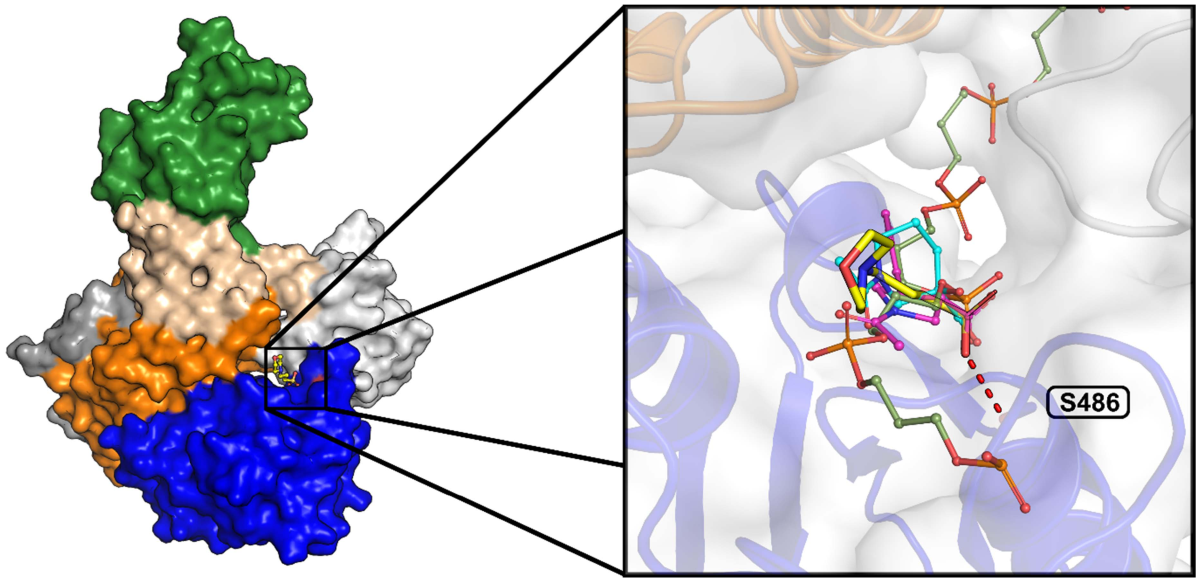

Figure S1 Binding site of MOPS at the 5′-RNA binding site in SARS-CoV-2 NSP13. The left panel shows NSP13 in surface representation, colour-coded by domains as in Figure 1, with MOPS depicted in ball-and-stick format (yellow). The right panel provides a close-up of the 5′ end of the RNA-binding channel of NSP13 (PDB entry 9I4V), highlighting that MOPS forms a hydrogen bond via its sulfonic acid group with Ser486 (red dashed lines). Additionally, MOPS is superimposed on RNA-bound NSP13 (PDB entry 7KRO) and fragment-bound structures (PDB entries 5RLZ and 5RMM), illustrating that its sulfonic acid group overlaps with the phosphate of the RNA (smudge, representing only the phosphate backbone for clarity) and the carboxyl groups of fragments VWM (cyan) and VXG (magenta).

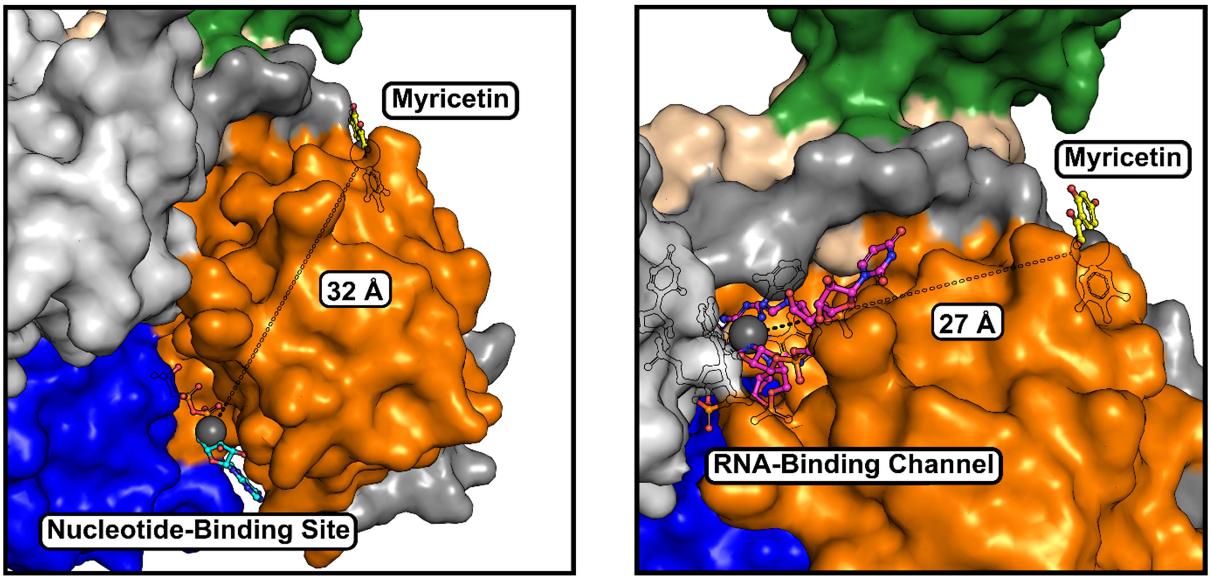

(a) *(b)*

Figure S2 Distances between myricetin's binding site, the nucleotide-binding site, and the RNA-binding channel in SARS-CoV-2 NSP13. The structures of NSP13 bound to myricetin (PDB entry 9I1S), AMP-PNP (PDB entry 7NN0), and RNA (PDB entry 7CXM) were aligned to illustrate the spatial relationships between myricetin's binding site, nucleotide-binding site and RNA-binding channel. NSP13 is shown in surface representation, coloured according to Figure 1, with myricetin (yellow), AMP-PNP (cyan), and RNA (magenta) in ball-and-stick format. Distances from myricetin's centre-of-mass to those of AMP-PNP and RNA (grey spheres) were measured, revealing 32 Å to the nucleotide-binding site (a) and 27 Å to the RNA-binding channel (b).

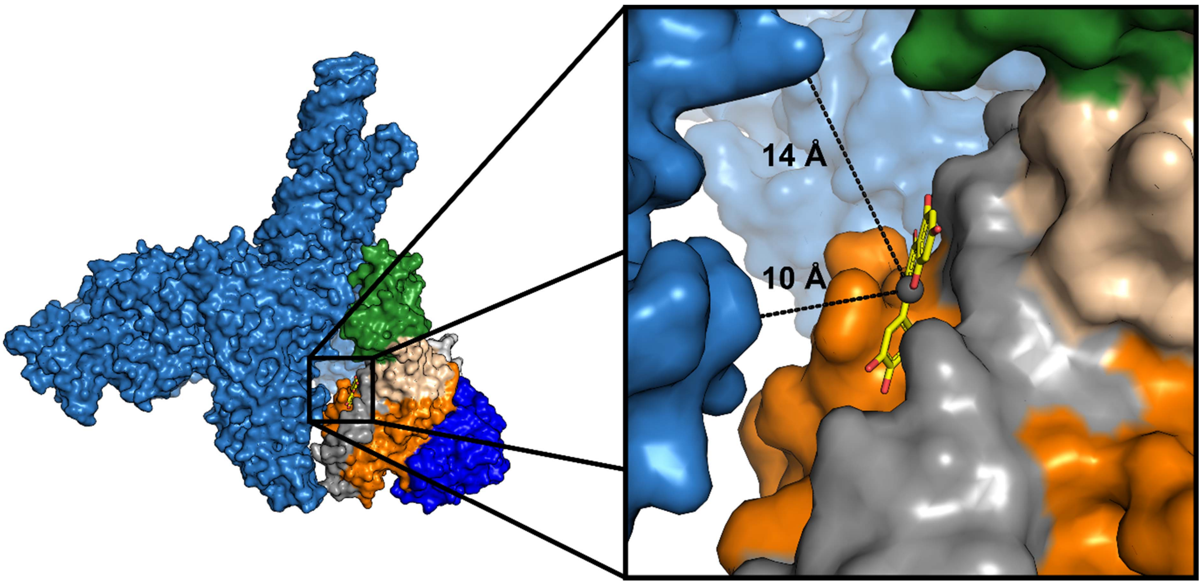

Figure S3 Myricetin's binding site accessibility within the replicase-transcriptase complex (RTC) of SARS-CoV-2. The left panel shows the RTC (PDB entry 6XEZ) in surface representation, with NSP13 color-coded by domain as in Figure 1, while the rest of the RTC is coloured in light blue. The right panel provides a zoomed-in view of myricetin's binding site, highlighting its position within the RTC. Distances to nearby structural features (10 Å and 14 Å) demonstrate that the site remains exposed and accessible, supporting its potential as a target for inhibitor binding during replication of SARS-CoV-2.

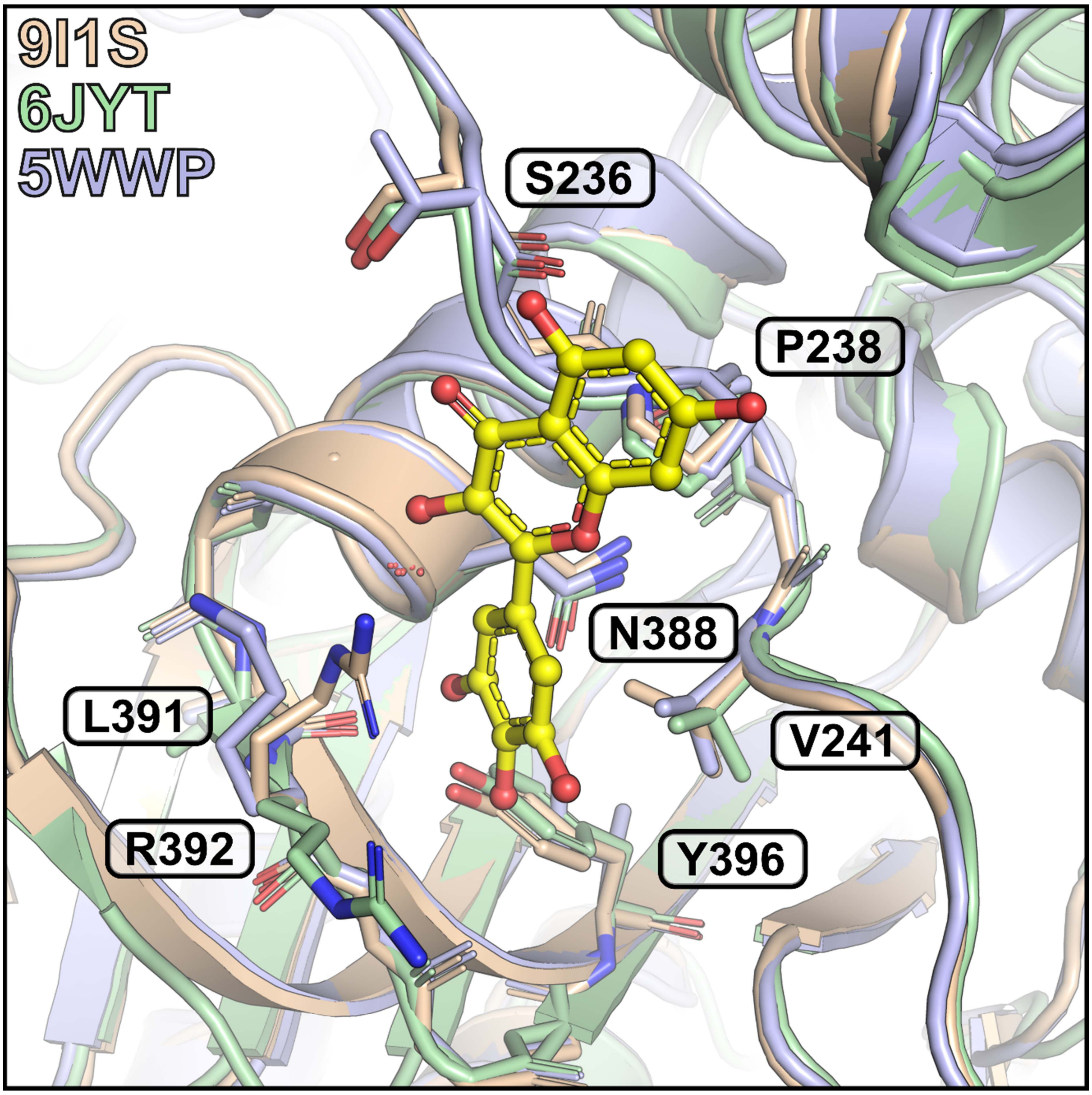

Figure S4 The structural alignment of NSP13 from SARS-CoV-2, SARS-CoV, and MERS-CoV. The aligned structures of SARS-CoV-2 NSP13 (PDB entry 9I1S, chain B, wheat), SARS-CoV NSP13 (PDB entry 6JYT, chain B, green), and MERS-CoV NSP13 (PDB entry 5WWP, chain B, blue) are shown in cartoon representation, highlighting the position of myricetin's binding site in SARS-CoV-2 NSP13. Myricetin is depicted in ball-and-stick format and coloured yellow. Residues interacting with myricetin in SARS-CoV-2 NSP13 are displayed as sticks and labelled.

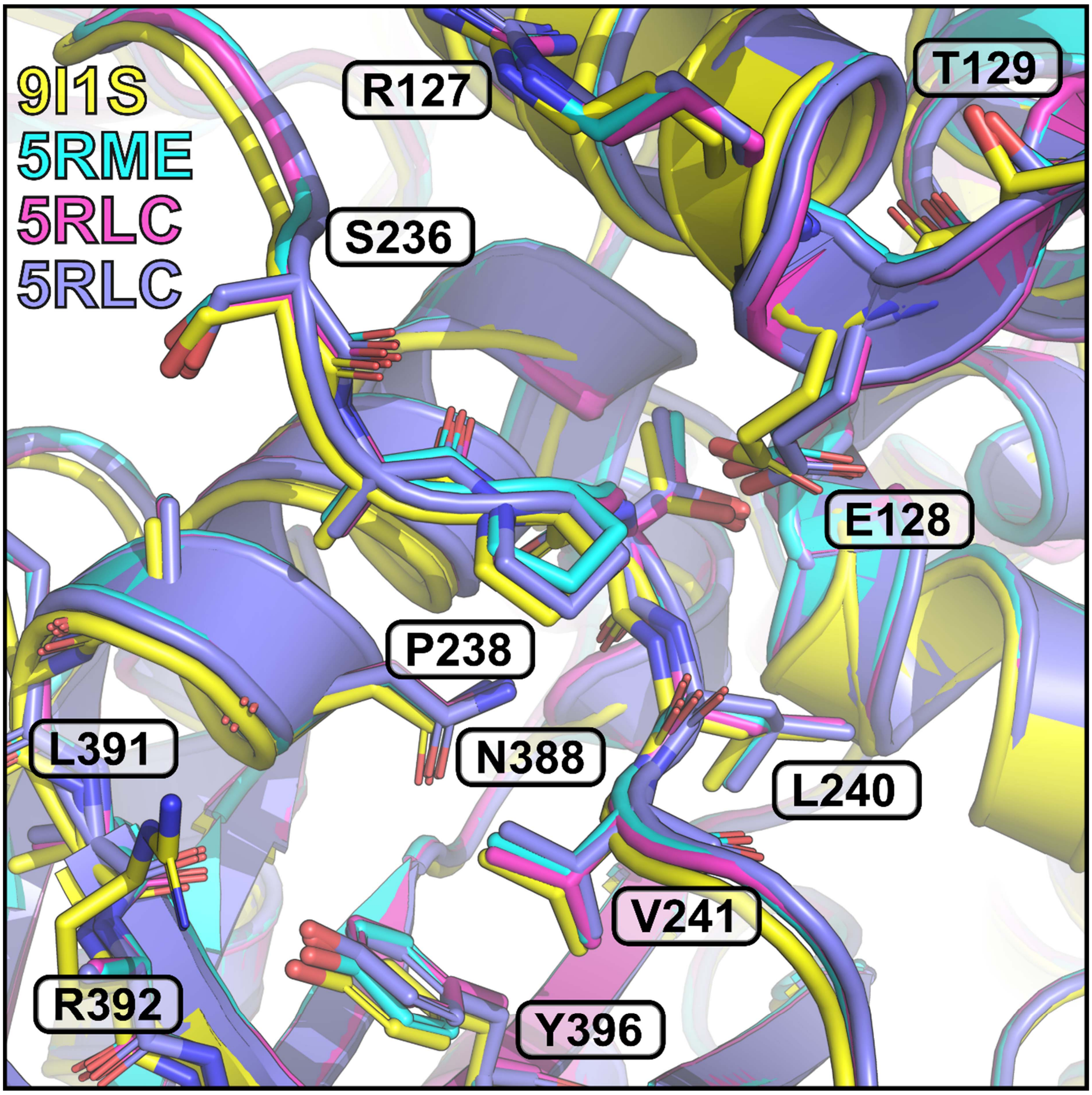

Figure S5 The structural comparison of myricetin's binding site in myricetin-bound and fragment-bound SARS-CoV-2 NSP13. Myricetin-bound NSP13 structure (PDB entry 9I1S, chain B, yellow) is shown alongside fragment-bound NSP13 structures from Newman et al. (2021): RYM-bound (PDB entry 5RME, chain B, cyan), VVM-bound (PDB entry 5RLC, chain B, magenta), and VVY-bound (PDB entry 5RLD, chain B, blue). All structures are shown in cartoon representation, with interacting residues labelled and displayed as sticks. Superimposed structures reveal no significant differences in the binding site, with pairwise alignments yielding RMSD values below 0.32 Å. These findings validate the use of the myricetin-bound structure alone for docking with HYBRID and Gnina.

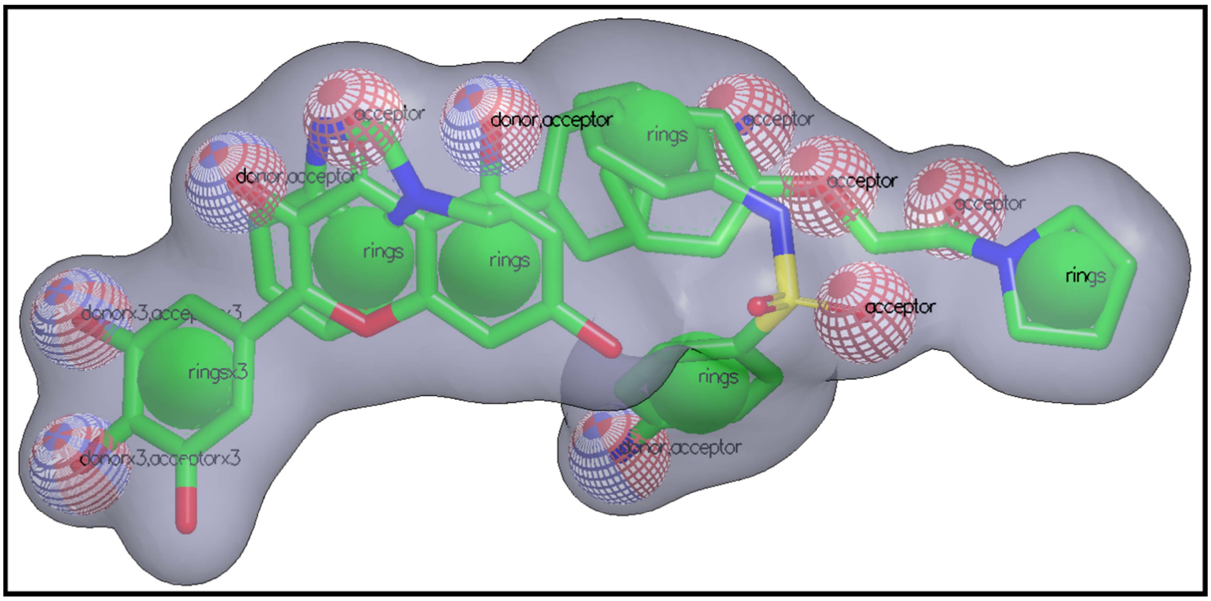

Figure S6 ROCS query for virtual screening of potential inhibitors targeting SARS-CoV-2 NSP13. The query is based on the myricetin-bound NSP13 complex, incorporating the binding conformations of myricetin and overlapping fragments (RYM, VVM, and VVY), represented as their combined shape (grey). Key chemical features, including hydrogen bond donors (blue spheres), hydrogen bond acceptors (red spheres), and aromatic rings (green surfaces), capture conserved interactions with NSP13. Features of myricetin's energetically favourable pyrogallol moiety are upweighted threefold. This query guided ligand-based virtual screening to identify pyrogallol-based compounds integrating myricetin's and fragment-derived characteristics for binding to NSP13.

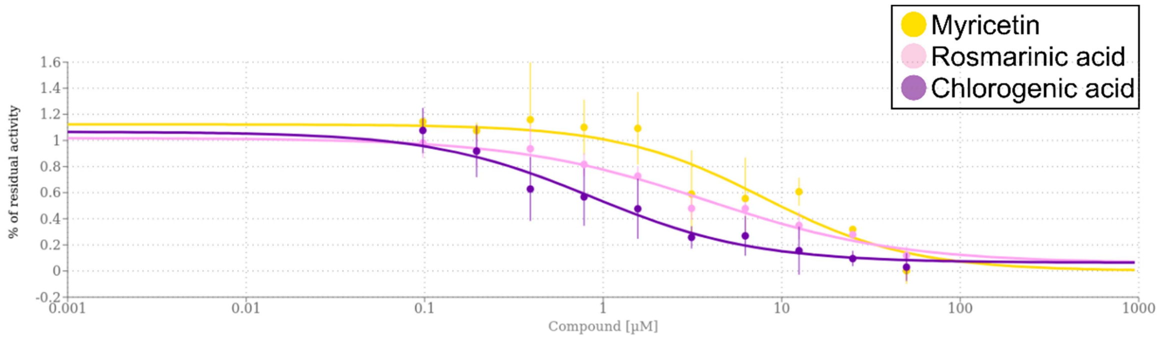

(a)

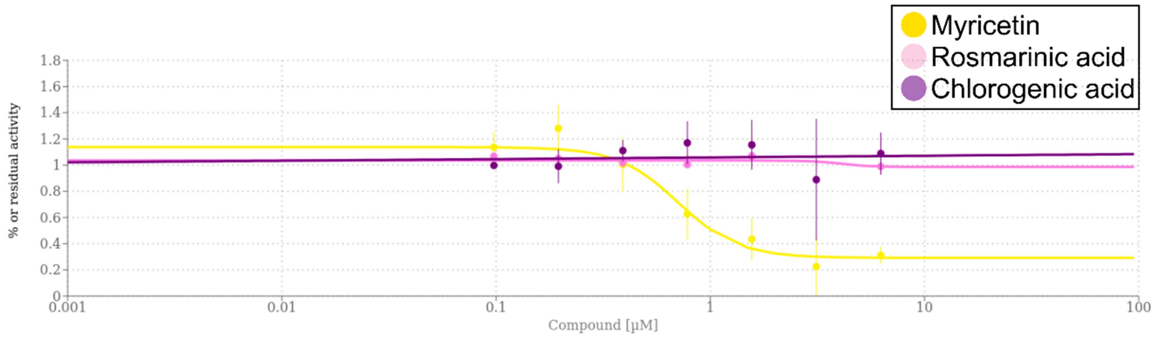

(b)

Figure S7 Dose-dependent inhibition of SARS-CoV-2 NSP13 by myricetin, rosmarinic acid, and chlorogenic acid. (a) Residual unwinding activity and (b) residual ATPase activity of NSP13 were measured at varying concentrations of myricetin (yellow), rosmarinic acid (pink), and chlorogenic acid (purple). Data represent the mean and standard deviation of triplicate measurements, background-corrected and normalised. IC_{50} values were calculated using an online IC_{50} calculator (AAT Bioquest, 2024). All three compounds inhibit NSP13's unwinding activity, with chlorogenic acid being the most potent (IC_{50} = 0.87 µM), followed by rosmarinic acid (IC_{50} = 3.83 µM) and myricetin (IC_{50} = 7.91 µM). Only myricetin inhibits ATPase activity, with an IC_{50} of 0.72 µM.

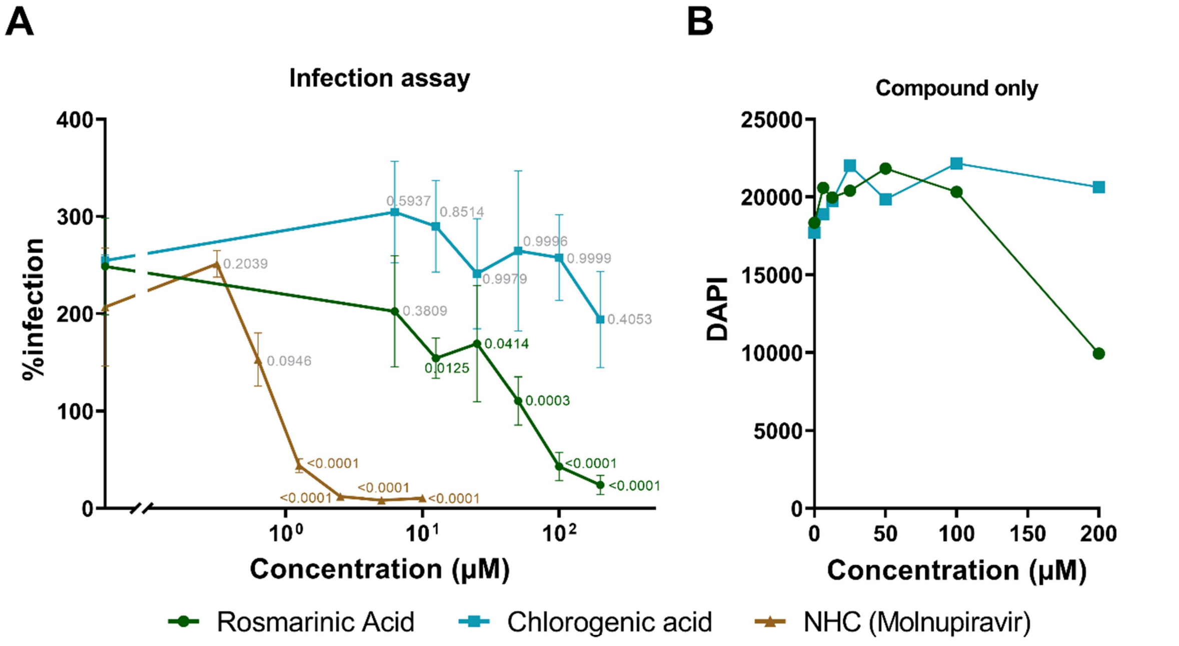

Figure S8 Evaluation of the antiviral activity of rosmarinic acid, chlorogenic acid, and NHC (the active compound derived from the drug Molnupiravir). (a) Infection assay showing the percentage of infected Vero E6 cells treated with varying concentrations of rosmarinic acid (green), chlorogenic acid (blue), and NHC (orange). Rosmarinic acid reduced infection at higher concentrations, with an IC_{50} of 59.31 µM, while chlorogenic acid showed no significant effect. NHC, used as a positive control, had an IC_{50} of 0.7454 µM. Data represent the mean of triplicate measurements and p-values from Dunnett's multiple comparison test are indicated. (b) DAPI signal of uninfected cells treated with rosmarinic acid and chlorogenic acid, showing a reduction at 200 µM rosmarinic acid. Figures were generated using GraphPad Prism 10 for Windows (GraphPad Software LLC, San Diego, CA, USA).
